# Supplementary material for: Causes of admissions and in-hospital mortality among patients admitted to critical care units in primary and secondary hospitals in Vietnam in 2018: a multicentre retrospective study
Source: BMJ Open. 2022 Jun 12;12(6):e061638. doi: 10.1136/bmjopen-2022-061638 (PMC9196179; doi:10.1136/bmjopen-2022-061638)
Supplement: Supplementary data [file bmjopen-2022-061638supp001.pdf]

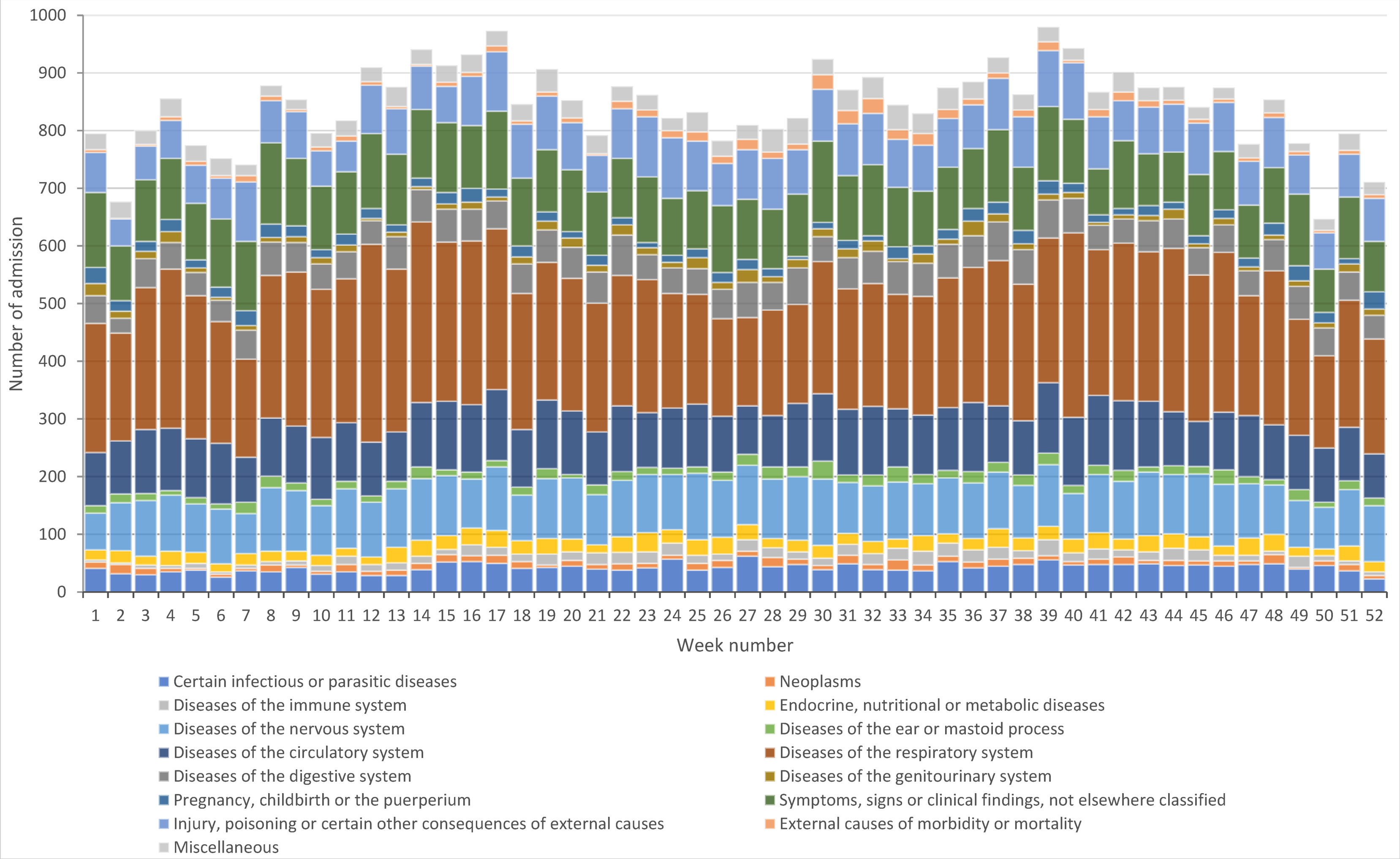

Supplementary Figure 1. Pattern of admissions over months.

Supplementary Table 1. Mortality and years of life lost (YLL) grouped by ICD-11 chapter codes

Diagnosis	Number and proportion of death cases	In-hospital mortality (n,%)	Total YLL75	Proportion of YLLs75	YLL75 per 1000 patients	Length of stay to death (median, IQR)
**Diseases of the respiratory system**	367 (21.8%)	4.8%	4506000	25.9%	813	6 (3-13)
**Diseases of the nervous system**	324 (19.3%)	8.1%	3994000	14.0%	1432	2 (2-6)
**Symptoms, signs or clinical findings, not elsewhere classified**	241 (14.3%)	9.4%	5141000	9.8%	2476	2 (1-4)
**Injury, poisoning or certain other consequences of external causes**	210 (12.5%)	8.4%	5846000	8.8%	2557	2 (1-2)
**Certain infectious or parasitic diseases**	188 (11.2%)	14.0%	3218000	5.8%	3056	4 (2-10)
**Diseases of the circulatory system**	112 (6.7%)	2.8%	817000	13.4%	314	3 (1-5)
**Diseases of the digestive system**	83 (4.9%)	3.9%	1221000	7.1%	683	3 (2-8)
**Neoplasms**	59 (3.5%)	17.3%	809000	1.3%	3385	5 (2-9)
**Miscellaneous**	31 (1.8%)	3.5%	963000	3.1%	1259	2 (1-5)
**Endocrine, nutritional or metabolic diseases**	22 (1.3%)	2.4%	217000	3.3%	276	2 (1-3)
**Diseases of the immune system**	20 (1.2%)	3.3%	890000	2.2%	1532	2 (1-2)
**Diseases of the genitourinary system**	13 (0.8%)	3.0%	168000	1.5%	487	5 (2-11)
**External causes of morbidity or mortality**	8 (0.5%)	1.8%	223000	1.6%	556	3.5 (1.5-4.5)
**Diseases of the ear or mastoid process**	2 (0.1%)	0.4%	87000	1.8%	192	1 (1-1)
**Pregnancy, childbirth or the puerperium**	1 (0.1%)	0.7%	35000	0.5%	241	1 (1-1)
**Total**	1681 (100.0%)	5.9%	28135000	100.0%	1287	3 (2-7)

Supplementary Table 2. The number of deaths by ICD 4-character codes

	Causes of death	N (%)
**CHAPTER 01 Certain infectious or parasitic diseases**	Viral intestinal infections, unspecified	1 (0.06%)
	Gastroenteritis or colitis without specification of infectious agent	1 (0.06%)
	Tuberculosis of the respiratory system	2 (0.12%)
	Bacterial cellulitis, erysipelas and lymphangitis	1 (0.06%)
	Other specified pyogenic bacterial infection of skin and subcutaneous tissue	1 (0.06%)
	Tetanus	1 (0.06%)
	Bacterial infection of unspecified site	1 (0.06%)
	Viral encephalitis not elsewhere classified	2 (0.12%)
	Viral meningitis not elsewhere classified	1 (0.06%)
	Infectious abscess of the central nervous system	1 (0.06%)
	Dengue fever, unspecified	1 (0.06%)
	Acute viral hepatitis	4 (0.24%)
	Viral hepatitis, unspecified	2 (0.12%)
	Phaeohyphomycosis	1 (0.06%)
	Sepsis without septic shock	23 (1.37%)
	Sepsis with septic shock	126 (7.50%)
	Infection, unspecified	19 (1.13%)
**CHAPTER 02 Neoplasms**	Primary neoplasms of brain	6 (0.36%)
	Malignant haematopoietic neoplasms without further specification	1 (0.06%)
	Malignant neoplasms of other or ill-defined sites in the lip, oral cavity or pharynx	2 (0.12%)
	Malignant neoplasms of oesophagus	5 (0.30%)
	Malignant neoplasms of stomach	2 (0.12%)
	Malignant neoplasms of colon	5 (0.30%)
	Malignant neoplasm of pancreas	1 (0.06%)
	Malignant neoplasms of liver or intrahepatic bile ducts	11 (0.65%)
	Malignant neoplasms of gallbladder	1 (0.06%)
	Malignant neoplasms of bronchus or lung	10 (0.59%)
	Malignant neoplasms of thymus	1 (0.06%)
	Malignant neoplasms of heart, mediastinum or non-mesothelioma of pleura	2 (0.12%)
	Malignant neoplasms of breast, unspecified	3 (0.18%)
	Malignant neoplasms of prostate	1 (0.06%)
	Malignant neoplasms of bladder	1 (0.06%)
	Malignant neoplasms of thyroid gland	1 (0.06%)
	Malignant neoplasm metastasis in lung	4 (0.24%)
	Carcinoma in situ of bladder	1 (0.06%)
	Benign osteogenic tumours	1 (0.06%)
**CHAPTER 03 Diseases of the blood or blood-forming organs**	Anaemia due to chronic disease	1 (0.06%)
	Anaemias or other erythrocyte disorders, unspecified	8 (0.48%)
	Anaphylaxis	9 (0.54%)

	Causes of death	N (%)
**CHAPTER 04 Diseases of the immune system**	Allergic or hypersensitivity conditions of unspecified type	11 (0.65%)
**CHAPTER 05 Endocrine, nutritional or metabolic diseases**	Type 1 diabetes mellitus	3 (0.18%)
	Type 2 diabetes mellitus	3 (0.18%)
	Hypoglycaemia without associated diabetes	5 (0.30%)
	Increased secretion of glucagon	1 (0.06%)
	Protein deficiency	1 (0.06%)
	Hyperosmolality or hypernatraemia	1 (0.06%)
	Acidosis	1 (0.06%)
	Hyperkalaemia	1 (0.06%)
	Hypokalaemia	1 (0.06%)
	Disorders of fluid, electrolyte or acid-base balance, unspecified	5 (0.30%)
**CHAPTER 06 Mental, behavioural or neurodevelopmental disorders**	Developmental learning disorder	1 (0.06%)
	Dissociative disorders, unspecified	4 (0.24%)
	Disorders due to use of dissociative drugs including ketamine and phencyclidine [PCP]	1 (0.06%)
	Delirium	1 (0.06%)
	Mental, behavioural or neurodevelopmental disorders, unspecified	2 (0.12%)
**CHAPTER 08 Diseases of the nervous system**	Seizure due to acute causes	2 (0.12%)
	Types of seizures	11 (0.65%)
	Epilepsy or seizures, unspecified	3 (0.18%)
	Intracerebral haemorrhage	133 (7.91%)
	Subarachnoid haemorrhage	3 (0.18%)
	Intracranial haemorrhage, unspecified	32 (1.90%)
	Cerebral ischaemic stroke	72 (4.28%)
	Stroke not known if ischaemic or haemorrhagic	61 (3.63%)
	Late effects of cerebrovascular disease	2 (0.12%)
	Myelitis	1 (0.06%)
	Motor neuron disease	1 (0.06%)
	Hereditary sensory or autonomic neuropathy	1 (0.06%)
	Increased intracranial pressure	1 (0.06%)
	Disorders of the meninges excluding infection	1 (0.06%)
**CHAPTER 10 Diseases of the ear or mastoid process**	Disorders of vestibular function	2 (0.12%)
**CHAPTER 11 Diseases of the circulatory system**	Essential hypertension	8 (0.48%)
	Hypertensive heart disease	1 (0.06%)
	Hypotension, unspecified	5 (0.30%)
	Angina pectoris	1 (0.06%)
	Acute myocardial infarction	36 (2.14%)
	Pulmonary thromboembolism	1 (0.06%)
	Pulmonary hypertension	1 (0.06%)
	Pulmonary heart disease or diseases of pulmonary circulation, unspecified	5 (0.30%)
	Pericardial effusion	1 (0.06%)
	Mitral valve stenosis with insufficiency	1 (0.06%)

	Causes of death	N (%)
	Myocarditis	1 (0.06%)
	Conduction disorders	1 (0.06%)
	Ventricular tachyarrhythmia	2 (0.12%)
	Cardiac arrhythmia, unspecified	5 (0.30%)
	Congestive heart failure	3 (0.18%)
	Heart failure, unspecified	36 (2.14%)
	Acute arterial occlusion	1 (0.06%)
	Aortic aneurysm or dissection	1 (0.06%)
	Diseases of arteries or arterioles, unspecified	1 (0.06%)
	Deep vein thrombosis	1 (0.06%)
**CHAPTER 12 Diseases of the respiratory system**	Acute tonsillitis	1 (0.06%)
	Acute laryngitis or tracheitis	1 (0.06%)
	Acute upper respiratory infections of multiple and unspecified sites	1 (0.06%)
	Bronchitis	2 (0.12%)
	Chronic obstructive pulmonary disease	52 (3.09%)
	Asthma	8 (0.48%)
	Bronchiectasis	1 (0.06%)
	Pneumonia	198 (11.78%)
	Abscess of lung or mediastinum	2 (0.12%)
	Lung infections, unspecified	1 (0.06%)
	Pneumonitis due to solids and liquids	1 (0.06%)
	Respiratory conditions due to inhalation of chemicals, gases, fumes or vapours	3 (0.18%)
	Acute respiratory distress syndrome	2 (0.12%)
	Pulmonary oedema	11 (0.65%)
	Pneumothorax	2 (0.12%)
	Haemothorax	1 (0.06%)
	Pleural effusion	1 (0.06%)
	Respiratory failure	79 (4.70%)
**CHAPTER 13 Diseases of the digestive system**	Gastro-oesophageal reflux disease	1 (0.06%)
	Vascular disorders of the oesophagus	3 (0.18%)
	Gastritis	4 (0.24%)
	Gastric ulcer	5 (0.30%)
	Obstruction of small intestine	7 (0.42%)
	Appendicitis	3 (0.18%)
	Obstruction of large intestine	1 (0.06%)
	Polyp of large intestine	1 (0.06%)
	Infectious liver disease	1 (0.06%)
	Acute or subacute hepatic failure	2 (0.12%)
	Hepatic fibrosis or cirrhosis	15 (0.89%)
	Autoimmune liver disease	1 (0.06%)
	Certain specified diseases of liver	17 (1.01%)
	Cholelithiasis	2 (0.12%)
	Cholangitis	2 (0.12%)
	Acute pancreatitis	8 (0.48%)
	Peritonitis	6 (0.36%)
	Inguinal hernia	1 (0.06%)
	Indeterminate colitis	1 (0.06%)

	Causes of death	N (%)
	Diseases of the digestive system, unspecified	2 (0.12%)
**CHAPTER 14 Diseases of the skin**	Lichen planus	1 (0.06%)
	Skin disease of unspecified nature	1 (0.06%)
**CHAPTER 16 Diseases of the genitourinary system**	Pyonephrosis	1 (0.06%)
	Acute kidney failure	1 (0.06%)
	Chronic kidney disease	7 (0.42%)
	Kidney failure, unspecified	2 (0.12%)
	Urinary tract infection, site not specified	2 (0.12%)
**CHAPTER 18 Pregnancy, childbirth or the puerperium**	Complications following abortion, ectopic or molar pregnancy	1 (0.06%)
**CHAPTER 19 Certain conditions originating in the perinatal period**	Sepsis of foetus or newborn	1 (0.06%)
	Respiratory failure of newborn	1 (0.06%)
	Transitory disorders of carbohydrate metabolism specific to foetus or newborn	1 (0.06%)
	Certain specified transitory neonatal electrolyte or metabolic disturbances	1 (0.06%)
**CHAPTER 20 Developmental anomalies**	Congenital hydrocephalus	1 (0.06%)
	Congenital anomaly of a ventricle or the ventricular septum	1 (0.06%)
	Structural developmental anomalies of gallbladder, bile ducts or liver	1 (0.06%)
**CHAPTER 21 Symptoms, signs or clinical findings, not elsewhere classified**	Symptoms, signs or clinical findings involving consciousness	22 (1.31%)
	Abnormalities of heart beat	2 (0.12%)
	Cardiac arrest	48 (2.86%)
	Abnormalities of breathing	2 (0.12%)
	Haemorrhage from throat	1 (0.06%)
	Haemoptysis	2 (0.12%)
	Respiratory arrest	23 (1.37%)
	Abdominal or pelvic pain	4 (0.24%)
	Nausea or vomiting	1 (0.06%)
	Clinical manifestations of the digestive system	56 (3.33%)
	Cachexia	1 (0.06%)
	Fever of other or unknown origin	4 (0.24%)
	Haemorrhage, not elsewhere classified	1 (0.06%)
	Shock	60 (3.57%)
	Syncope and collapse	1 (0.06%)
	Other sudden death, cause unknown	1 (0.06%)
	Other ill-defined and unspecified causes of mortality	9 (0.54%)
	Multi organ failure	3 (0.18%)
**CHAPTER 22 Injury, poisoning or certain other consequences of external causes**	Injury of cranial nerves	1 (0.06%)
	Intracranial injury	65 (3.87%)
	Superficial injury of thorax	2 (0.12%)
	Injury of blood vessels at abdomen, lower back or pelvis level	1 (0.06%)
	Injury of intra-abdominal organs	5 (0.30%)
	Traumatic amputation of forearm	1 (0.06%)

	Causes of death	N (%)
	Fracture of femur	1 (0.06%)
	Superficial injury of ankle or foot	1 (0.06%)
	Crushing injuries involving multiple body regions	1 (0.06%)
	Unspecified multiple injuries	28 (1.67%)
	Burn of wrist or hand	1 (0.06%)
	Burns of external body surface, other specified site	1 (0.06%)
	Burns of multiple body regions	1 (0.06%)
	Harmful effects of drugs, medicaments or biological substances, not elsewhere classified	57 (3.39%)
	Harmful effects of or exposure to noxious substances, chiefly nonmedicinal as to source, not elsewhere classified	30 (1.78%)
	Effects of other specified external causes	3 (0.18%)
	Certain early complications of trauma, not elsewhere classified	11 (0.65%)
**CHAPTER 23 External causes of morbidity or mortality**	Unintentional exposure to or harmful effects of pesticides	1 (0.06%)
	Intentional self-harm by threat to breathing by suffocation from object covering mouth or nose	1 (0.06%)
	Intentional self-harm by and exposure to other and unspecified drug, medicament and biological substance	3 (0.18%)
	Intentional self-harm by exposure to or harmful effects of pesticides	1 (0.06%)
	Intentional self-harm by exposure to or harmful effects of other or unspecified substances chiefly nonmedicinal as to so	1 (0.06%)
	Exposure to or harmful effects of undetermined intent of other or unspecified drugs, medicaments or biological substance	1 (0.06%)
**CHAPTER 24 Factors influencing health status or contact with health services**	Surgical or postsurgical states, unspecified	3 (0.18%)
	Personal history of malignant neoplasm	1 (0.06%)
