# Supplementary material for: Causes of admissions and in-hospital mortality among patients admitted to critical care units in primary and secondary hospitals in Vietnam in 2018: a multicentre retrospective study
Source: BMJ Open. 2022 Jun 12;12(6):e061638. doi: 10.1136/bmjopen-2022-061638 (PMC9196179; doi:10.1136/bmjopen-2022-061638)
Supplement: Author's manuscript [file bmjopen-2022-061638.draft_revisions.pdf]

BMJ Open

BMJ Open is committed to open peer review. As part of this commitment we make the peer review history of every article we publish publicly available.

When an article is published we post the peer reviewers' comments and the authors' responses online. We also post the versions of the paper that were used during peer review. These are the versions that the peer review comments apply to.

The versions of the paper that follow are the versions that were submitted during the peer review process. They are not the versions of record or the final published versions. They should not be cited or distributed as the published version of this manuscript.

BMJ Open is an open access journal and the full, final, typeset and author-corrected version of record of the manuscript is available on our site with no access controls, subscription charges or pay-per-view fees (http://bmjopen.bmj.com).

If you have any questions on BMJ Open's open peer review process please email info.bmjopen@bmj.com

BMJ Open

Causes of admissions and in-hospital mortality among patients admitted to critical care units in primary and secondary hospitals in Vietnam: a multicentre retrospective study in 2018

Journal:	*BMJ Open*
Manuscript ID	bmjopen-2022-061638
Article Type:	Original research
Date Submitted by the Author:	09-Feb-2022
Complete List of Authors:	Dat, Vu Quoc; Hanoi Medical University, Department of Infectious Diseases<br>Linh, Bui Thi Khanh; Hanoi Medical University, Department of Infectious Diseases<br>Kim, Giang; Hanoi Medical University
Keywords:	Public health < INFECTIOUS DISEASES, Epidemiology < TROPICAL MEDICINE, Adult intensive & critical care < INTENSIVE & CRITICAL CARE, Epidemiology < INFECTIOUS DISEASES

SCHOLARONE™
Manuscripts

I, the Submitting Author has the right to grant and does grant on behalf of all authors of the Work (as defined in the below author licence), an exclusive licence and/or a non-exclusive licence for contributions from authors who are: i) UK Crown employees; ii) where BMJ has agreed a CC-BY licence shall apply, and/or iii) in accordance with the terms applicable for US Federal Government officers or employees acting as part of their official duties; on a worldwide, perpetual, irrevocable, royalty-free basis to BMJ Publishing Group Ltd ("BMJ") its licensees and where the relevant Journal is co-owned by BMJ to the co-owners of the Journal, to publish the Work in this journal and any other BMJ products and to exploit all rights, as set out in our licence.

The Submitting Author accepts and understands that any supply made under these terms is made by BMJ to the Submitting Author unless you are acting as an employee on behalf of your employer or a postgraduate student of an affiliated institution which is paying any applicable article publishing charge ("APC") for Open Access articles. Where the Submitting Author wishes to make the Work available on an Open Access basis (and intends to pay the relevant APC), the terms of reuse of such Open Access shall be governed by a Creative Commons licence – details of these licences and which Creative Commons licence will apply to this Work are set out in our licence referred to above.

Other than as permitted in any relevant BMJ Author's Self Archiving Policies, I confirm this Work has not been accepted for publication elsewhere, is not being considered for publication elsewhere and does not duplicate material already published. I confirm all authors consent to publication of this Work and authorise the granting of this licence.

For peer review only

Causes of admissions and in-hospital mortality among patients admitted to critical care units in primary and secondary hospitals in Vietnam: a multicentre retrospective study in 2018

Running Title: Burden of disease in patients admitted to critical care units in Vietnam

Vu Quoc Dat[1]*, Bui Thi Khanh Linh[1], Kim Bao Giang[2]

[1] Department of Infectious Diseases, Hanoi Medical University, Hanoi, Vietnam, [2] Institute for Preventive Medicine and Public Health, Hanoi Medical University, Hanoi, Vietnam

*Corresponding author: Vu Quoc Dat MD, PhD, Department of Infectious Diseases, Hanoi Medical University. E-mail: datvq@hmu.edu.vn/quocdat181@yahoo.com. Postal address: No 1 Ton That Tung street, Dong Da district, Hanoi, Vietnam.

Keywords: International Classification of Diseases, ICD-11, emergency, diagnosis, burden of disease, mortality, Vietnam

For peer review only

Abstract

Objective

The goal of this study was to describe the burden of disease and in-hospital mortality among patients admitted to the critical care units (CCUs) in Vietnam.

Design

Retrospective study.

Setting

The whole 1-year data of admissions to CCUs were collected from 34 hospitals from January to December 2018.

Participants

A total of 44,013 episodes of admission to CCUs were analysed.

Primary outcome

We used ICD-11 codes to assess the primary diagnosis associated with admissions and in-hospitals mortality. Years of life lost (YLL) measure was further used to estimate the burden of disease.

Results

The 0-5 year and ≥70 year age groups accounted for 14.8 % and 26.1% of all admissions, respectively. The most common diagnoses were diseases of the respiratory system (27.8%), followed by unclassified symptoms, signs or clinical findings (13%), and diseases of the circulatory system (12.2%). Among 28,311 patients with available outcome data, 1,681 individuals (5.9%) died during the hospitalization. The in-hospital mortality rate increased with age, from 2.8% in under 5 years old age group to 23.1% in over 90-year age group. Diseases of the respiratory system was the leading causes of death in term of number of deaths (21.8% of all deaths). Diagnosis of sepsis was associated with the highest in-hospital mortality (36.8%). The overall YLL under the age of 75 were 1287 per 1000 patients.

Conclusions

CCUs in Vietnam faced wide differences in the burden of diseases. Sufficient infrastructure, adequate multidisciplinary training are essential to ensure the appropriate response to the current needs of population.

For peer review only - http://bmjopen.bmj.com/site/about/guidelines.xhtml

Strengths and limitations of this study

- This is the first multicentre study with a large population and data collection for the whole year of 2018 among patients admitted to critical care units in Vietnam before the COVID-19 pandemic
- This is the first report on years of life lost due to premature death in critical care units in provincial and district hospitals in Vietnam.
- The main limitation was that we relied on the available diagnosis description and diagnostic coding, data quality may vary between hospitals due to the accurate clinical diagnosis of disease depends on doctor's knowledge and skills, available laboratory investigations and diagnostic imaging procedures.

For peer review only

Introduction

Emergency care is an essential service of the hospitals which serves as a first point of contact to provide immediate treatment for individual with acute illness and life-threatening needs. Over 50% of mortality and over 40% of all morbidities worldwide can be contributed by emergency medical conditions [1]. In May 2019, the 63rd World Health Assembly recognized that existing data do not provide adequate support for effective planning and resource allocation for emergency care and urged member states to identify the local acute illness burden for improving quality of emergency care [2].

The International Statistical Classification of Diseases and Related Health Problems (often referred to as the International Classification of Diseases, ICD) has been used in more than 120 countries to monitor the incidence and prevalence of diseases [3]. Vietnam has implemented the ICD since 1998 as a tool for reporting morbidity, cause of death and billing for health insurance in hospitals. The burden of disease in Vietnam has changed rapidly over the past 2 decades with a marked shift toward the predominance of non-communicable diseases. Between 2000 and 2017, the share of communicable disease has reduced a half (24% to 14%), the share of non-communicable disease has increased almost 10% (63% to 74%) while the share of injuries still remained stable at around 12-13% (all measured in the disability-adjusted life year, DALY) [4]. However, the research specifically measuring the burden of diseases in emergency departments (EDs) or intensive care capable EDs (referred to as critical care units or CCUs) in Vietnam has been limited. Recently, some studies on the pattern of hospitalization, the causes of death or the hospital death certification have been performed [5-7], but none of these studies has provided a comprehensive description of the patterns of diagnosis or measured the premature death by the years of life lost (YLL) metric in patients with admission to CCUs in the country.

The lack of infrastructure and human resource brings significant challenges to the delivery of critical care in low- and middle-income countries (LMICs)[8]. The emergence of COVID-19 has put further pressure on the critical care system in these countries that already struggling with the high burden of admission to the CCUs in the pre-COVID-19 era. An understanding of the conditions that involve an admission to CCU will allows policymakers and planners to implement interventions that are effective in the national contexts to meet the care quality needs of the rapidly expanding aging populations and to prevent the

For peer review only - http://bmjopen.bmj.com/site/about/guidelines.xhtml

disruption of the critical care services in the pandemic. Our aim was to describe the frequency of diagnostic categories, in-hospital mortality, cause of death and the burden of premature mortality measuring by YLL in CCUs in Vietnam in 2018.

Methods

Study design and participants

This was a retrospective study of all episodes of CCU admissions to selected provincial and district hospitals in Vietnam from 1st January to 31st December 2018. We purposely selected 5 out of 63 provinces to represent 5 geographical areas in Vietnam. In each province, we invited all CCUs in hospitals in the catchment area.

Data were collected from the electronic hospital management system, including patient's demographic information, diagnosis, dates of admission, length of stay and outcome at discharge. Diagnosis of individual patients in each episode of admission were originally recorded in the ICD, 10th revision (ICD-10) codes. Because the ICD, 11th revision (ICD-11) came into effect in January 2022, for the monitoring of any change in the pattern of diseases responsible for admission in the future, all ICD-10 codes were translated to the ICD-11 codes (version 05/2021) using WHO mapping tables with the correspondence between ICD-10 and ICD-11 codes and link the ICD-11 codes with their corresponding associated descriptive labels. Two independent investigators (VQD and BTKL) manually reviewed any non-unique code or discrepancies in mapping between ICD-10 and ICD-11 which were eventually resolved by the consensus of 2 reviewers with the reference to the majority of code assignments by treating doctors.

The outcomes from the hospitalization were defined as death in patients who died in the hospital or was going home to die imminently after hospital discharge. Deterioration was defined as any worsening of clinical conditions by the treating doctor's judgment at the time of discharge.

Statistical Analysis

For the analysis of pattern of admission, we included all episodes of admission. Consequently, one patient might have multiple episodes of admission to CCUs during a year. For each episode, the primary diagnosis which was defined as the main reason for CCU admission was counted. The primary diagnosis for that episode may be the same or

For peer review only - http://bmjopen.bmj.com/site/about/guidelines.xhtml

different from the previous episode of admission. Individual patients were identified within the datasets by matching on full name, gender, date of birth and insurance numbers when they were available). The in-hospital mortality was calculated by dividing the number of deaths by the total number of individual patients.

Years of life lost (YLLs) is a measure of premature mortality which is calculated from the number of deaths at each age multiplied by a standard life expectancy for the age at which death occurs [9]. Vietnam's standard life expectancy at birth in 2019 for both sexes were 73.7 years old [10] and to compare with other studies, we considered premature death were deaths under 75 years old. We also calculated the average YLL by dividing total of YLL by the number of premature death and the crude YLL rate by dividing total of YLL by the total number of patients aged under 75 years (expressed per 1000 persons).

We presented categorical variables as frequencies and percentages, while continuous variables as medians and interquartile ranges [IQR]. We used the chi-square test or Fisher's exact test to compare two binomial proportions. Statistical analyses were performed using SPSS version 27 (IBM, Armonk, NY, USA). Significant differences were considered if two-sided P values were < 0.05.

Patient and public involvement

Patients and the public were not involved in the design, recruitment or conduct of the study.

Results

Between 1 January 2018 and 31 December 2018, there were 44,013 admission episodes to CCUs from 34 hospitals in 5 provinces (namely Hanoi, Thai Nguyen, Ha Nam, Kon Tum and Can Tho), corresponding to 38534 unique patients. There were 3,647 (9.5%) patients with two or more admission in the study year (**Error! Reference source not found.**). The median age was 53 years (interquartile range [IQR]) 25-70) and included 53.4% male. The majority of CCU admissions in this study (31,569, 71.7%) were at the district hospitals. The median length of stay was 5 (IQR 2-8) days. A total of 1,764/36,518 admissions (4.8%) had a length of stay of more than 14 days. The hospitalization outcome was available for 28,311/38,534 (73.5%) patients. Overall survival to hospital discharge was 94.1% (26,630/28,311).

For peer review only - http://bmjopen.bmj.com/site/about/guidelines.xhtml

Admission diagnosis in critical care units

Of 44,013 episodes of admission to CCUs, the most common diagnosis were respiratory diseases (12,255 episodes, 27.8%) in which, 38.7% was certain lower respiratory tract diseases (blockL1-CA2) and 34.4% was lung infections (blockL1-CA4), followed by unclassified symptoms, signs or clinical findings (5,712 episodes or 13.0%) (51.8% was of digestive system or abdomen, blockL1-MD8), circulatory system diseases (5,380 episodes, 12.2%) (51,2% was hypertensive diseases, blockL1-BA0), diseases of the nervous system (5,019 episodes, 11.4%) (85.4% was cerebrovascular diseases, blockL1-8B0) and injury, poisoning or consequences of external causes (4,186 episodes or 9.5%) (39.7% was harmful effect off substances, blockL1-NE6). The percentage of diagnosis in episodes of CCU admission varied by age groups (**Error! Reference source not found.**). The number of admissions peaked in children from 5 years old and younger and was lowest in the 5-10 year age group (**Error! Reference source not found.**). The respiratory disease was the leading cause of admission in the under 10 year age group (64% of admissions) and age groups of 61 years and older (29% of admissions) (**Error! Reference source not found.**)

The number of admission in all study hospitals fluctuated across months but mostly driven by the admission by respiratory diseases (**Error! Reference source not found.**). The proportions of admission to CCUs were significantly higher in provincial hospitals than in district hospital in regard to diseases of the nervous system (18.7% vs 8.5%, p<0.001), certain infectious or parasitic diseases (8.9% vs 3.4%, p<0.001), injury/poisoning (10.3% vs 9.2%, p=0.00), endocrine, nutritional or metabolic diseases (3.6% vs 2.2%, p<0.001) and neoplasms (2% vs 0.7%, p<0.001) (**Error! Reference source not found.Error! Reference source not found.**).

Outcome of hospitalization

The median length of stay for all admissions was 5 days (IQR 2-8). Among 28,311 patients with available outcome data and, 1,681/28,311 (5.9%) died. The median age of death was 68 years (IQR 47-86) and 63.3% of death were in men. Men had significantly higher in-hospital mortality rate than women (3.8% vs. 2.6%, p<0.001). The median duration of hospital stay for the survivors and death cases was 4 (IQR:2-8) and 3 (IQR: 2–7), respectively. More than three quarters of deaths (1,290/1,681 or 76.7%) occurred in provincial hospitals. The all-cause in-hospital mortality rates by age groups and mortality

For peer review only - http://bmjopen.bmj.com/site/about/guidelines.xhtml

rate for selected groups of diagnosis are presented in **Error! Reference source not found.** and **Error! Reference source not found.**. The most common causes of death by ICD-11 block codes and chapter codes are showed in **Error! Reference source not found.** and **Error! Reference source not found.**, respectively.

For peer review only

For peer review only - http://bmjopen.bmj.com/site/about/guidelines.xhtml

Table 1. The top 20 causes of death in terms of years of life lost (YLL) and numbers of deaths by ICD-11 block level 1 codes

Diagnosis	Number and proportion of death cases	In-hospital mortality (n,%)	Total YLL75	Proportion of YLLs75	YLL75 per 1000 patients	Length of stay to death (median, IQR)
Cerebrovascular diseases	303 (18.0%)	8.7%	3211000	12.3%	1380	3 (2-6)
Miscellaneous	259 (15.4%)	2.3%	5686000	38.2%	614	2 (1-6)
Lung infections	201 (12.0%)	7.2%	2875000	9.5%	1440	7 (3-15)
Sepsis	149 (8.9%)	36.8%	1991000	2.6%	8127	4 (2-11)
Harmful effects of substances	87 (5.2%)	6.1%	3534000	5.0%	2544	1 (1-2)
Respiratory failure	79 (4.7%)	18.1%	365000	1.6%	1714	9 (5-15)
General symptoms, signs or clinical findings	67 (4.0%)	13.5%	1507000	2.2%	3712	2 (1-4.5)
Injuries to the head	66 (3.9%)	15.6%	1140000	1.6%	3167	2 (2-4)
Certain lower respiratory tract diseases	63 (3.7%)	2.1%	443000	10.2%	217	4 (2-10)
Symptoms, signs or clinical findings of the digestive system or abdomen	61 (3.6%)	5.5%	994000	4.0%	1062	3 (2-6)
Malignant neoplasms, except primary neoplasms of lymphoid, haematopoietic, central nervous system or related	50 (3.0%)	18.0%	587000	1.0%	3073	6 (3-9)
Symptoms, signs or clinical findings of the circulatory system	50 (3.0%)	31.8%	1060000	0.7%	8217	2 (1-6)
Heart failure	39 (2.3%)	8.4%	280000	1.6%	1466	3 (1-5)
Ischaemic heart diseases	37 (2.2%)	5.2%	78000	2.5%	158	3 (1-5)
Diseases of liver	36 (2.1%)	11.2%	549000	1.1%	1861	2.5 (1.5-7.5)
Injuries involving multiple body regions	29 (1.7%)	28.2%	439000	0.4%	6362	2 (1-6)
Symptoms, signs or clinical findings of the respiratory system	28 (1.7%)	8.4%	706000	1.1%	3017	2 (1-4)
Mental or behavioural symptoms, signs or clinical findings	22 (1.3%)	13.3%	363000	0.8%	2771	2 (1-2)
Allergic or hypersensitivity conditions	20 (1.2%)	3.3%	890000	2.2%	1537	2 (1-2)
Infection, unspecified	19 (1.1%)	31.7%	741000	0.2%	14529	2 (2-4)
Epilepsy or seizures	16 (1.0%)	5.0%	696000	1.1%	2275	1.5 (1-2)
Total	1681 (100.0%)	5.9%	28135000	100.0%	1287	3 (2-7)

For peer review only

For peer review only - http://bmjopen.bmj.com/site/about/guidelines.xhtml

The in-hospital mortality in patients over 75 years was 662/6,140 (10.8%). In total, 28,135,000 years were lost among patient admitted to the study CCUs. This yielded an overal YLL under age of 75 years of 1,287 per 1,000 people. **Error! Reference source not found.** presents the top 10 causes of death by ICD-11 block codes. Cerebrovascular diseases rank the first in the number of deaths (303/1,681 or 18%) and the eleven when measuring in in-hospital mortality (8.7%) while sepsis ranked as the first specified cause of death in terms of in-hospital mortality (36.8%) and ranked fourth in term of death counts.

Discussion

This study presents for the first time, data to quantify the burden of diseases in CCUs, for the most common reasons of admission and the in-hospital mortality of patients admitted to CCUs in Vietnam. Few studies on admission to the CCUs in lower-middle income countries have been reported earlier.

Burden of diseases in Vietnam has changed significantly in the past 20 years [4]. The communicable diseases burden (measured in disability-adjusted life years) decreased rapidly from 37% in 1990 to 14% in 2017. However, in the critical care settings, our study shows that the infectious diseases remain a major emergency medical condition and are the prime cause of morbidity and mortality in terms of number of admission and in terms of YLL.

Our findings on the difference distribution of diseases in CCUs in provincial and district hospitals reflected the variation of healthcare services and capacity of clinical management. Provincial hospitals in Vietnam are the secondary hospitals which do not only treat referred patients from lower level of care (district hospitals or commune centres) but also admit patients from the community. Additionally, patient can directly self-refer to higher level of hospitals which resulting in under-utilization of district hospitals and over crowding in provincial hospitals, especially for given specialities which requires higher levels of skills and capacity.

Our findings are partly aligned with previous reports in all cause mortality in both sexes and all ages showing that cardiovascular (ischaemic heart disease, stroke), respiratory diseases and injuries were the significant challenge. WHO's report in 2019 showed that ischaemic heart disease, stroke, neonatal conditions, chronic obstructive pulmonary disease (COPD) and lower respiratory infections were the top 5 causes of death in term of death

For peer review only - http://bmjopen.bmj.com/site/about/guidelines.xhtml

counts in LMICs [11]. At the global level in 2019, the top three diseases with highest burden (measuring in DALY) were neonatal disorder (32.9% of global DALYs), lower respiratory infections (11% of global DALYs) and diarrhoeal diseases (9.3% of global DALYs) in children younger than 10 years and were ischemic heart diseases (16.2% of global DALY), stroke (13% of global DALY) and COPD (8.5% of global DALY) in patients from 75 years and older [12]. In Vietnam, stroke (164.8 deaths per 100,000), ischaemic heart disease (95.3 deaths per 100,000), COPD (37.2 deaths per 100,000) and diabetes mellitus (35.1 deaths per 100,000) and road injuries (30.5 deaths per100,000) were in the top-five rankings for total number of death in 2019 [13]. Sepsis and harmful effects of substances were not listed in the top 5 cause of death in the general population, but we found that these were important conditions in emergency care settings.

The overall mortality in our study were higher than previous report. In a systematic review of data from 65 emergency departments in 59 LMICs countries between 2013 and 2014, the median mortality was 1.8% (IQR 0.5-5.1%), highest in sub-Saharan Africa (median 3.4%, IQR: 0.5–6.3%) and lowest in South Asia, East Asia & Pacific (0.3, IQR 0.2–0.8%) [14].

In our study, patients aged 75 years or older presented 18.9% of all admission at CCUs despite the fact that they comprised 3.2% of general population [15]. The percentage of population older than 75 years in Vietnam is expected to increase to 4.2% by 2030 and 6.6% by 2040 [15]. This will increase the demand for critical care resources and requires further evaluation to guide healthcare decision-making and changes in organizational models in CCU and delivery of critical care services during a pandemic.

The design of multicentre study, large population and data collection for the whole year of 2018 are the strengths of our study. The major limitations related to the diagnosis description and diagnostic coding by doctors in study hospitals. Data quality of diagnosis may vary between hospitals due to the accurate clinical diagnosis of disease depends on doctor's knowledge and skills, available laboratory investigations and diagnostic imaging procedures. However, there are differences in the capacity of clinical management and use of diagnostics between provincial and district hospitals in the country [16]. In Vietnam, treating doctors are responsible for assigning the ICD diagnostic code to each patient discharged from the hospital. The complex nature of ICD coding, patient's condition in

For peer review only - http://bmjopen.bmj.com/site/about/guidelines.xhtml

critical care settings and the lack of coding experience pose further challenges for the accuracy and reliability of the diagnosis given across hospitals.

Conclusion

Our study indicates a high burden of respiratory and infectious diseases and variation of needs of emergency care by age groups and levels of care among patients admitted to CCUs in provincial and district hospitals in Vietnam. These findings support the need to provide multidisciplinary team training for clinicians in CCUs and the future studies should further assess the emergency and critical care capacity needs, ability of the healthcare setting to provide the emergency services and the quality of care (including minimal set of indicators) and gaps to mobilize the resources required for live saving during a pandemic.

Data availability statement

Data are available upon reasonable request.

Ethics statements

Patient consent for publication

Not applicable

Ethics approval

This study was approved by the institutional review board (IRB) in the Hanoi Medical University (59/GCN-DDNCYSH-DHYHN).

Funding statement

This work was supported by Viet Nam - WHO Country Office. The funder of the study had no role in study design, data collection, data analysis, data interpretation, or writing of the report.

Competing interests

We declare no competing interest.

Contributors

VQD conceived and designed the study. VQD analysed the data and wrote the first draft of the report. VQD, BTKL and KBG involved to the acquisition and interpretation of data. All authors contributed to and approved the final report.

For peer review only - http://bmjopen.bmj.com/site/about/guidelines.xhtml

Acknowledgments

We are very grateful to the patients and staff at the participating hospitals in Ha Noi, Thai Nguyen, Ha Nam, Kon Tum and Can Tho. We would like to thank Drs. Satoko Otsu and Vu Quang Hieu (World Health Organization, Vietnam Country Office) for their tireless support and contribution to strengthening the capacity for clinicans in Vietnam and thank Dr. Nguyen The Hung (National Hospital for Tropical Diseases) for his initial support in the implementation of this study.

REFERENCES

1. Razzak J, Usmani MF, Bhutta ZA. Global, regional and national burden of emergency medical diseases using specific emergency disease indicators: analysis of the 2015 Global Burden of Disease Study. *BMJ Glob Health* 2019;4(2):e000733. doi: 10.1136/bmjgh-2018-000733
2. World Health Assembly. Emergency care systems for universal health coverage: ensuring timely care for the acutely ill and injured. Geneva: World Health Organization, 2019.
3. Executive Board. International Classification of Diseases: International Statistical Classification of Diseases and Related Health Problems: update on the eleventh revision: report by the Director-General. Geneva: World Health Organization, 2018.
4. Elena Glinskaya, Annette De Kleine Feige, Vu Thi Lan Hoang, et al. Vietnam - Adapting to an Aging Society Washington, D.C: World Bank Group, 2021.
5. Nguyen NTT, Dien TM, Schindler C, et al. Childhood hospitalisation and related deaths in Hanoi, Vietnam: a tertiary hospital database analysis from 2007 to 2014. *BMJ open* 2017;7(7):e015260. doi: 10.1136/bmjopen-2016-015260
6. Hong TT, Phuong Hoa N, Walker SM, et al. Completeness and reliability of mortality data in Viet Nam: Implications for the national routine health management information system. *PloS one* 2018;13(1):e0190755. doi: 10.1371/journal.pone.0190755
7. Walton M, Harrison R, Chevalier A, et al. Improving hospital death certification in Vietnam: results of a pilot study of injury-related fatalities. *Inj Prev* 2018;24(5):324-31. doi: 10.1136/injuryprev-2017-042341
8. Murthy S, Leligdowicz A, Adhikari NK. Intensive care unit capacity in low-income countries: a systematic review. *PloS one* 2015;10(1):e0116949. doi: 10.1371/journal.pone.0116949
9. World Health Organization. WHO methods and data sources for global burden of disease estimates 2000-2019. In: Analytics DoDa, Division of Data AaDfI, eds. Geneva: World Health Organization,, 2020.
10. World Health Organization. Life expectancy and Healthy life expectancy. Data by country 2020 [updated 4th December 2021; cited 2022 6th January]. Available from: https://apps.who.int/gho/data/node.main.688 accessed 6th January 2022.
11. World Health Organization. The top 10 causes of death 2019 [updated 9th December 2020; cited 2021 11th December]. Available from: https://www.who.int/news-room/fact-sheets/detail/the-top-10-causes-of-death accessed 11th December 2021.
12. GBD 2019 Diseases and Injuries Collaborators. Global burden of 369 diseases and injuries in 204 countries and territories, 1990-2019: a systematic analysis for the Global Burden of Disease Study 2019. *Lancet* 2020;396(10258):1204-22. doi: 10.1016/S0140-6736(20)30925-9
13. World Health Organization. Global Health Estimates 2020: Deaths by Cause, Age, Sex, by Country and by Region, 2000-2019 2020 [cited 2021 13th December]. Available from: https://www.who.int/data/gho/data/themes/mortality-and-global-health-estimates/ghe-leading-causes-of-death accessed 13th December 2021.

For peer review only - http://bmjopen.bmj.com/site/about/guidelines.xhtml

14. Obermeyer Z, Abujaber S, Makar M, et al. Emergency care in 59 low- and middle-income countries: a systematic review. *Bull World Health Organ* 2015;93(8):577-86G. doi: 10.2471/BLT.14.148338
15. General Statistic Office, United Nations Population Fund. Vietnam population projection for the period 2019-2069. In: Office GS, ed. Hanoi, 2020.
16. Dat VQ, Hung NT, Giang BK, et al. Capacity and use of diagnostics and treatment for patients with severe acute respiratory infections in the pre-COVID-19 era in district and provincial hospitals in Viet Nam. *Western Pac Surveill Response J* 2021;12(4):1-9. doi: doi.org/10.5365/wpsar.2021.12.4.835

Figure Legend

Figure 1. Flowchart of CCU admission and outcome.

Figure 2. Diangosis on admission to CCU by age groups.

Figure 3. Number of admission and In-hospital mortality by age groups

Figure 4. Cause of admission to CCUs by level of care.

Supplementary materials

Supplementary Figure 1. Pattern of admissions over months.

Supplementary Table 1. Mortality and years of life lost (YLL) grouped by ICD-11 chapter codes

For peer review only

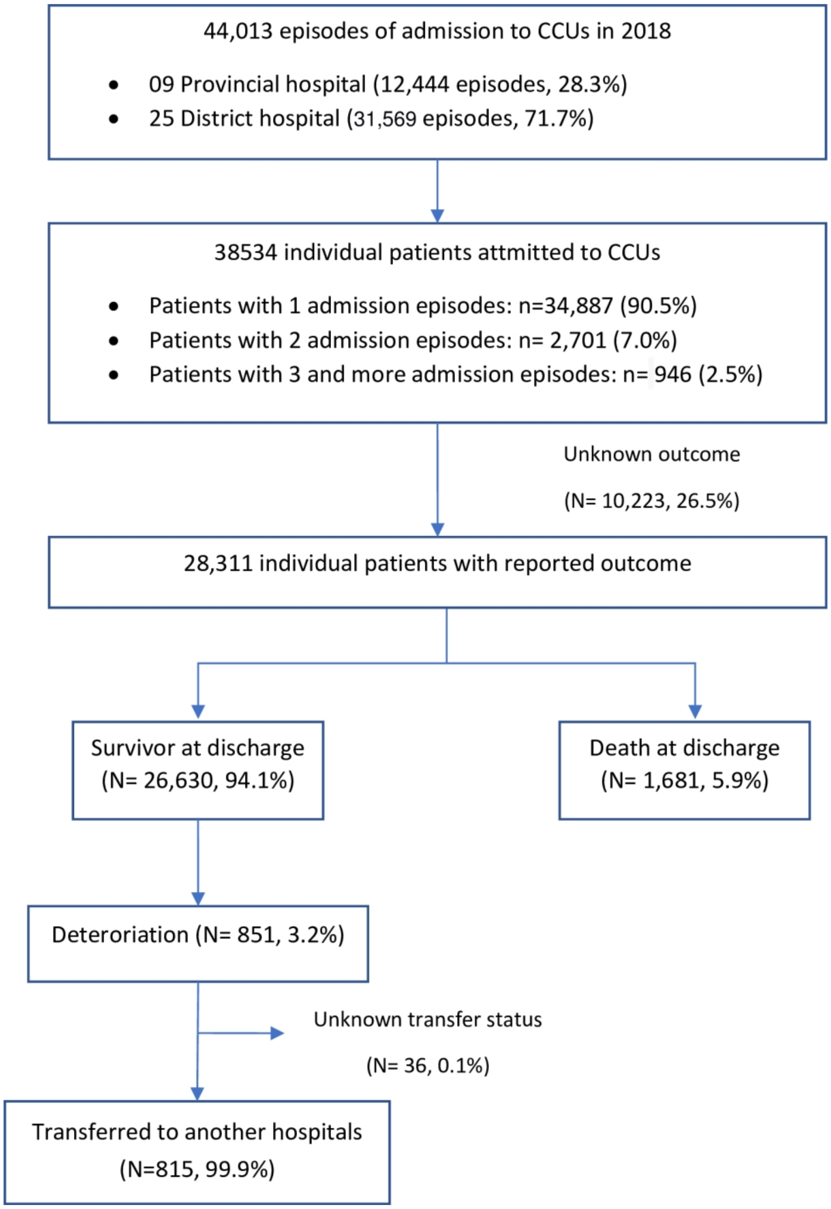

Figure 1. Flowchart of CCU admission and outcome.

127x185mm (300 x 300 DPI)

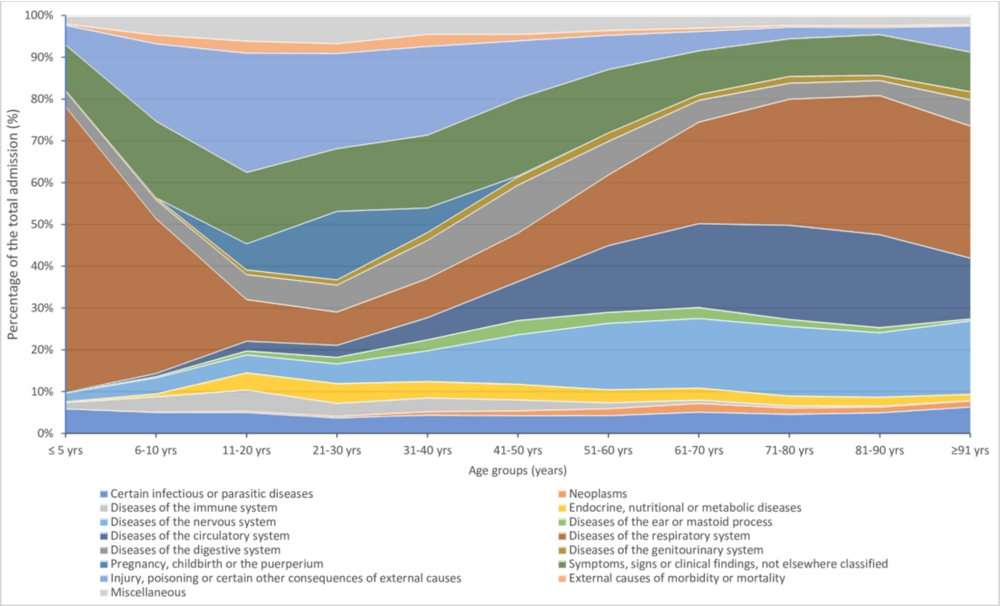

Figure 2. Diangosis on admission to CCU by age groups.

190x115mm (300 x 300 DPI)

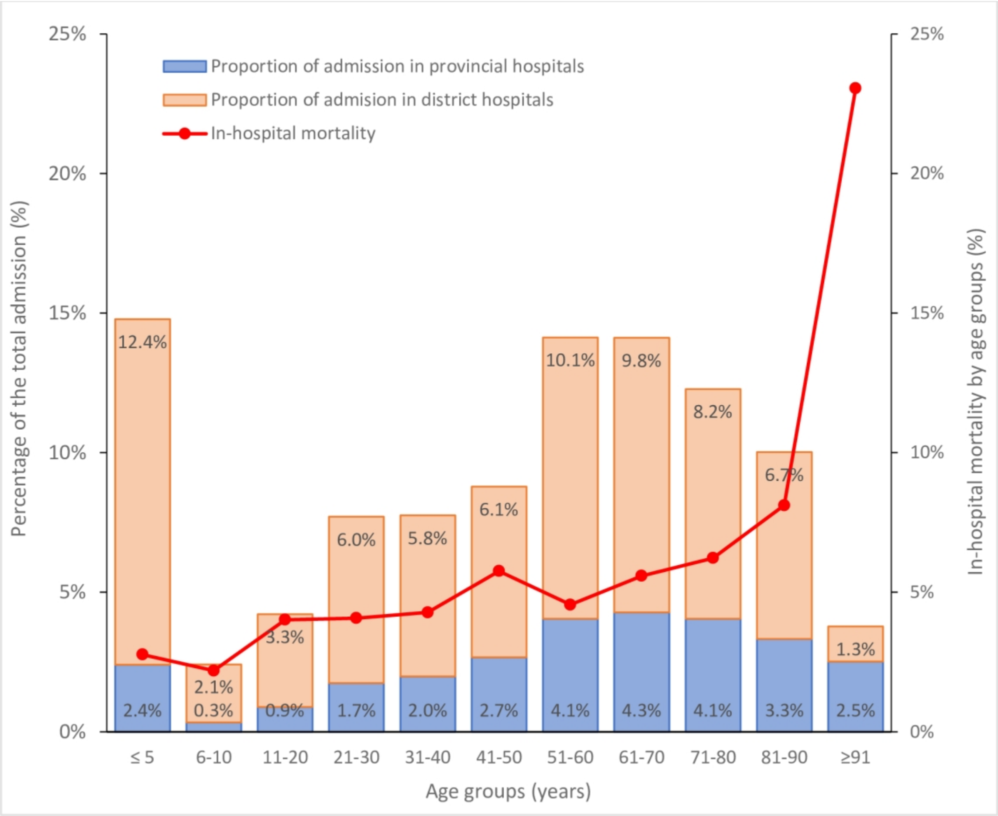

Figure 3. Number of admission and In-hospital mortality by age groups

159x129mm (300 x 300 DPI)

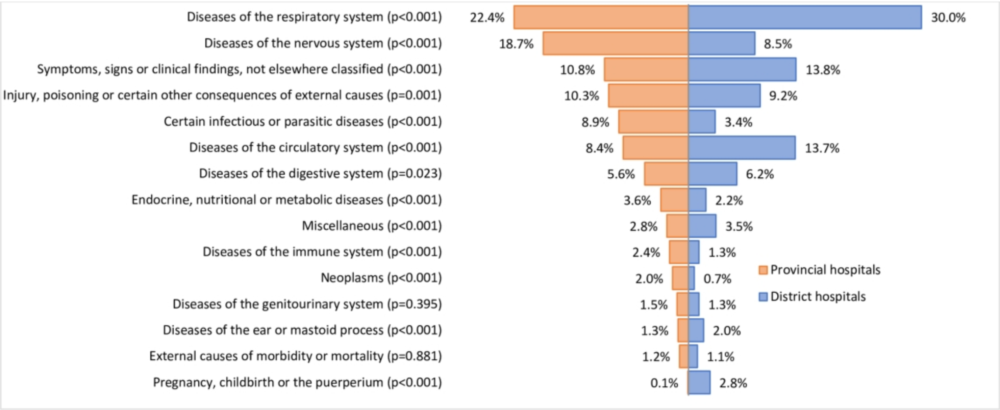

Figure 4. Cause of admission to CCUs by level of care.

190x77mm (300 x 300 DPI)

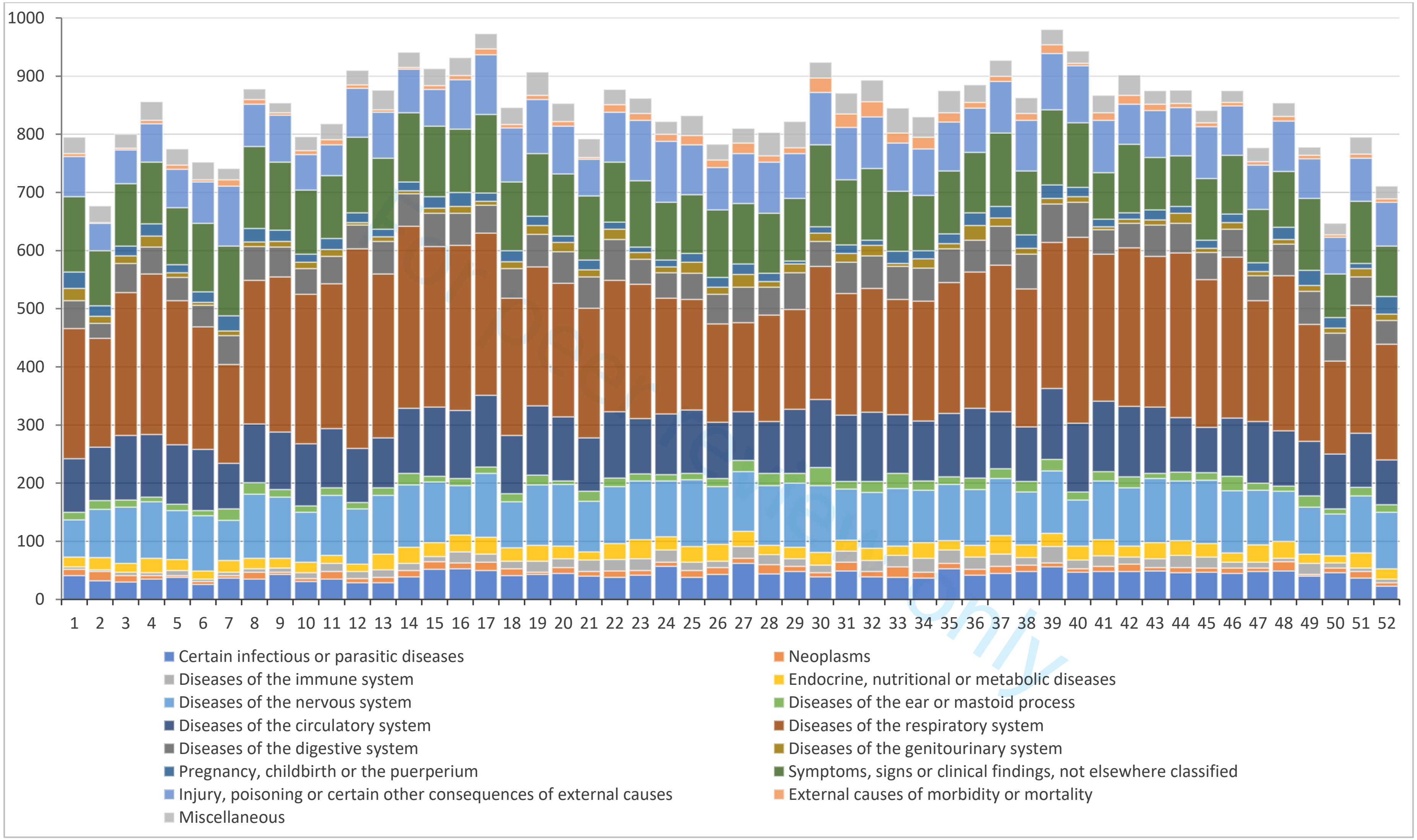

Supplementary Figure 1. Pattern of admissions over months.

Supplementary Table 1. Mortality and years of life lost (YLL) grouped by ICD-11 chapter codes

Diagnosis	Number and proportion of death cases	In-hospital mortality (n,%)	Total YLL75	Proportion of YLLs75	YLL75 per 1000 patients	Length of stay to death (median, IQR)
Diseases of the respiratory system	367 (21.8%)	4.8%	4506000	25.9%	813	6 (3-13)
Diseases of the nervous system	324 (19.3%)	8.1%	3994000	14.0%	1432	2 (2-6)
Symptoms, signs or clinical findings, not elsewhere classified	241 (14.3%)	9.4%	5141000	9.8%	2476	2 (1-4)
Injury, poisoning or certain other consequences of external causes	210 (12.5%)	8.4%	5846000	8.8%	2557	2 (1-2)
Certain infectious or parasitic diseases	188 (11.2%)	14.0%	3218000	5.8%	3056	4 (2-10)
Diseases of the circulatory system	112 (6.7%)	2.8%	817000	13.4%	314	3 (1-5)
Diseases of the digestive system	83 (4.9%)	3.9%	1221000	7.1%	683	3 (2-8)
Neoplasms	59 (3.5%)	17.3%	809000	1.3%	3385	5 (2-9)
Miscellaneous	31 (1.8%)	3.5%	963000	3.1%	1259	2 (1-5)
Endocrine, nutritional or metabolic diseases	22 (1.3%)	2.4%	217000	3.3%	276	2 (1-3)
Diseases of the immune system	20 (1.2%)	3.3%	890000	2.2%	1532	2 (1-2)
Diseases of the genitourinary system	13 (0.8%)	3.0%	168000	1.5%	487	5 (2-11)
External causes of morbidity or mortality	8 (0.5%)	1.8%	223000	1.6%	556	3.5 (1.5-4.5)
Diseases of the ear or mastoid process	2 (0.1%)	0.4%	87000	1.8%	192	1 (1-1)
Pregnancy, childbirth or the puerperium	1 (0.1%)	0.7%	35000	0.5%	241	1 (1-1)
Total	1681 (100.0%)	5.9%	28135000	100.0%	1287	3 (2-7)

For peer review only

STROBE Statement—Checklist of items that should be included in reports of ***cross-sectional studies***

	Item No	Recommendation	Page No
**Title and abstract**	1	(*a*) Indicate the study's design with a commonly used term in the title or the abstract	1
		(*b*) Provide in the abstract an informative and balanced summary of what was done and what was found	2
**Introduction**			
Background/rationale	2	Explain the scientific background and rationale for the investigation being reported	4
Objectives	3	State specific objectives, including any prespecified hypotheses	5
**Methods**			
Study design	4	Present key elements of study design early in the paper	5
Setting	5	Describe the setting, locations, and relevant dates, including periods of recruitment, exposure, follow-up, and data collection	5
Participants	6	(*a*) Give the eligibility criteria, and the sources and methods of selection of participants	5
Variables	7	Clearly define all outcomes, exposures, predictors, potential confounders, and effect modifiers. Give diagnostic criteria, if applicable	5
Data sources/ measurement	8*	For each variable of interest, give sources of data and details of methods of assessment (measurement). Describe comparability of assessment methods if there is more than one group	*6*
Bias	9	Describe any efforts to address potential sources of bias	9
Study size	10	Explain how the study size was arrived at	5
Quantitative variables	11	Explain how quantitative variables were handled in the analyses. If applicable, describe which groupings were chosen and why	NA
Statistical methods	12	(*a*) Describe all statistical methods, including those used to control for confounding	6
		(*b*) Describe any methods used to examine subgroups and interactions	6
		(*c*) Explain how missing data were addressed	Fig 11
		(*d*) If applicable, describe analytical methods taking account of sampling strategy	NA
		(*e*) Describe any sensitivity analyses	NA
**Results**			
Participants	13*	(a) Report numbers of individuals at each stage of study—eg numbers potentially eligible, examined for eligibility, confirmed eligible, included in the study, completing follow-up, and analysed	6
		(b) Give reasons for non-participation at each stage	6
		(c) Consider use of a flow diagram	
Descriptive data	14*	(a) Give characteristics of study participants (eg demographic, clinical, social) and information on exposures and potential confounders	6
		(b) Indicate number of participants with missing data for each variable of interest	6
Outcome data	15*	Report numbers of outcome events or summary measures	6

For peer review only

Main results	16	(*a*) Give unadjusted estimates and, if applicable, confounder-adjusted estimates and their precision (eg, 95% confidence interval). Make clear which confounders were adjusted for and why they were included	7
		(*b*) Report category boundaries when continuous variables were categorized	NA
		(*c*) If relevant, consider translating estimates of relative risk into absolute risk for a meaningful time period	NA
Other analyses	17	Report other analyses done—eg analyses of subgroups and interactions, and sensitivity analyses	7
**Discussion**			
Key results	18	Summarise key results with reference to study objectives	8
Limitations	19	Discuss limitations of the study, taking into account sources of potential bias or imprecision. Discuss both direction and magnitude of any potential bias	9
Interpretation	20	Give a cautious overall interpretation of results considering objectives, limitations, multiplicity of analyses, results from similar studies, and other relevant evidence	9
Generalisability	21	Discuss the generalisability (external validity) of the study results	9
**Other information**			
Funding	22	Give the source of funding and the role of the funders for the present study and, if applicable, for the original study on which the present article is based	10

*Give information separately for exposed and unexposed groups.

Note: An Explanation and Elaboration article discusses each checklist item and gives methodological background and published examples of transparent reporting. The STROBE checklist is best used in conjunction with this article (freely available on the Web sites of PLoS Medicine at http://www.plosmedicine.org/, Annals of Internal Medicine at http://www.annals.org/, and Epidemiology at http://www.epidem.com/). Information on the STROBE Initiative is available at www.strobe-statement.org.

For peer review only

For peer review only - http://bmjopen.bmj.com/site/about/guidelines.xhtml

BMJ Open

Causes of admissions and in-hospital mortality among patients admitted to critical care units in primary and secondary hospitals in Vietnam in 2018: a multicentre retrospective study

Journal:	*BMJ Open*
Manuscript ID	bmjopen-2022-061638.R1
Article Type:	Original research
Date Submitted by the Author:	02-May-2022
Complete List of Authors:	Dat, Vu Quoc; Hanoi Medical University, Department of Infectious Diseases<br>Linh, Bui Thi Khanh; Hanoi Medical University, Department of Infectious Diseases<br>Kim, Giang; Hanoi Medical University
**Primary Subject Heading**:	Emergency medicine
Secondary Subject Heading:	Infectious diseases, Health policy, Epidemiology
Keywords:	Public health < INFECTIOUS DISEASES, Epidemiology < TROPICAL MEDICINE, Adult intensive & critical care < INTENSIVE & CRITICAL CARE, Epidemiology < INFECTIOUS DISEASES

SCHOLARONE™
Manuscripts

I, the Submitting Author has the right to grant and does grant on behalf of all authors of the Work (as defined in the below author licence), an exclusive licence and/or a non-exclusive licence for contributions from authors who are: i) UK Crown employees; ii) where BMJ has agreed a CC-BY licence shall apply, and/or iii) in accordance with the terms applicable for US Federal Government officers or employees acting as part of their official duties; on a worldwide, perpetual, irrevocable, royalty-free basis to BMJ Publishing Group Ltd ("BMJ") its licensees and where the relevant Journal is co-owned by BMJ to the co-owners of the Journal, to publish the Work in this journal and any other BMJ products and to exploit all rights, as set out in our licence.

The Submitting Author accepts and understands that any supply made under these terms is made by BMJ to the Submitting Author unless you are acting as an employee on behalf of your employer or a postgraduate student of an affiliated institution which is paying any applicable article publishing charge ("APC") for Open Access articles. Where the Submitting Author wishes to make the Work available on an Open Access basis (and intends to pay the relevant APC), the terms of reuse of such Open Access shall be governed by a Creative Commons licence – details of these licences and which Creative Commons licence will apply to this Work are set out in our licence referred to above.

Other than as permitted in any relevant BMJ Author's Self Archiving Policies, I confirm this Work has not been accepted for publication elsewhere, is not being considered for publication elsewhere and does not duplicate material already published. I confirm all authors consent to publication of this Work and authorise the granting of this licence.

For peer review only

Causes of admissions and in-hospital mortality among patients admitted to critical care units in primary and secondary hospitals in Vietnam in 2018: a multicentre retrospective study

Running Title: Burden of disease in patients admitted to critical care units in Vietnam

Vu Quoc Dat[1]*, Bui Thi Khanh Linh[1], Kim Bao Giang[2]

[1] Department of Infectious Diseases, Hanoi Medical University, Hanoi, Vietnam, [2] Institute for Preventive Medicine and Public Health, Hanoi Medical University, Hanoi, Vietnam

*Corresponding author: Vu Quoc Dat MD, PhD, Department of Infectious Diseases, Hanoi Medical University. E-mail: datvq@hmu.edu.vn/quocdat181@yahoo.com. Postal address: No 1 Ton That Tung street, Dong Da district, Hanoi, Vietnam.

Keywords: International Classification of Diseases, ICD-11, emergency, diagnosis, burden of disease, mortality, Vietnam

For peer review only

For peer review only - http://bmjopen.bmj.com/site/about/guidelines.xhtml

Abstract

Objective

The goal of this study was to describe the burden of disease and in-hospital mortality among patients admitted to the critical care units (CCUs) in Vietnam.

Design

Retrospective study.

Setting

The whole 1-year data of admissions to CCUs were collected from 34 hospitals from January to December 2018.

Participants

A total of 44,013 episodes of admission to CCUs were analysed.

Primary outcome

We used ICD-11 codes to assess the primary diagnosis associated with admissions and in-hospitals mortality. Years of life lost (YLL) measure was further used to estimate the burden of disease.

Results

The 0-5 year and ≥70 year age groups accounted for 14.8 % (6,508/44,013) and 26.1% (11,480/44,013) of all admissions, respectively. The most common diagnoses were diseases of the respiratory system (27.8% or 12,255/44,013), followed by unclassified symptoms, signs or clinical findings (13% or 5,712/44,013), and diseases of the circulatory system (12.2% or 5,380/44,013). Among 28,311 patients with available outcome data, 1,681 individuals (5.9%) died during the hospitalization. The in-hospital mortality rate increased with age, from 2.8% (86/3,105) in under 5 years old age group to 23.1% (297/1,288) in over 90-year age group. Diseases of the respiratory system was the leading causes of death in term of number of deaths (21.8% or 367/1,681 of all deaths). Diagnosis of sepsis was associated with the highest in-hospital mortality (36.8%). The overall YLL under the age of 75 were 1287 per 1000 patients.

For peer review only - http://bmjopen.bmj.com/site/about/guidelines.xhtml

Conclusions

CCUs in Vietnam faced wide differences in the burden of diseases. Sufficient infrastructure, adequate multidisciplinary training are essential to ensure the appropriate response to the current needs of population.

Strengths and limitations of this study

- We conducted a large multicentre study on the pattern of admission and frequency of diagnosis in the critical care units in Vietnam, a lower-middle income country.
- We presented mortality, cause of admission and the first report on years of life lost due to premature death in critical care units in provincial and district hospitals in Vietnam.
- The major limitation related to the accuracy of the diagnosis and diagnostic coding in study hospitals.

For peer review only

Introduction

Emergency care is an essential service of the hospitals which serves as a first point of contact to provide immediate treatment for individual with acute illness and life-threatening needs. Over 50% of mortality and over 40% of all morbidities worldwide can be contributed by emergency medical conditions [1]. In May 2019, the 63rd World Health Assembly recognized that existing data do not provide adequate support for effective planning and resource allocation for emergency care and urged member states to identify the local acute illness burden for improving quality of emergency care [2].

The International Statistical Classification of Diseases and Related Health Problems (often referred to as the International Classification of Diseases, ICD) has been used in more than 120 countries to monitor the incidence and prevalence of diseases [3]. Vietnam has implemented the ICD since 1998 as a tool for reporting morbidity, cause of death and billing for health insurance in hospitals. The burden of disease in Vietnam has changed rapidly over the past 2 decades with a marked shift toward the predominance of non-communicable diseases. Between 2000 and 2017, the share of communicable disease has reduced a half (24% to 14%), the share of non-communicable disease has increased almost 10% (63% to 74%) while the share of injuries still remained stable at around 12-13% (all measured in the disability-adjusted life year, DALY) [4]. However, the research specifically measuring the burden of diseases in emergency departments (EDs) or intensive care capable EDs (referred to as critical care units or CCUs) in Vietnam has been limited. Recently, some studies on the pattern of hospitalization, the causes of death or the hospital death certification have been performed [5-7], but none of these studies has provided a comprehensive description of the patterns of diagnosis or measured the premature death by the years of life lost (YLL) metric in patients with admission to CCUs in the country.

The lack of infrastructure and human resource brings significant challenges to the delivery of critical care in low- and middle-income countries (LMICs)[8]. The emergence of COVID-19 has put further pressure on the critical care system in these countries that already struggling with the high burden of admission to the CCUs in the pre-COVID-19 era. An understanding of the conditions that involve an admission to CCU will allows policymakers and planners to implement interventions that are effective in the national contexts to meet the care quality needs of the rapidly expanding aging populations and to prevent the

For peer review only - http://bmjopen.bmj.com/site/about/guidelines.xhtml

disruption of the critical care services in the pandemic. Our aim was to describe the frequency of diagnostic categories, in-hospital mortality, cause of death and the burden of premature mortality measuring by YLL in CCUs in Vietnam in 2018.

Methods

Study design and participants

This was a retrospective study of all episodes of CCU admissions to selected provincial and district hospitals in Vietnam from 1st January to 31st December 2018. We purposely selected 5 out of 63 provinces to represent 5 geographical areas in Vietnam. In each province, we invited all CCUs in hospitals in the catchment area.

Data were collected from the electronic hospital management system, including patient's demographic information, diagnosis, dates of admission, length of stay and outcome at discharge. Diagnosis of individual patients in each episode of admission were originally recorded in the ICD, 10th revision (ICD-10) codes. Because the ICD, 11th revision (ICD-11) came into effect in January 2022, for the monitoring of any change in the pattern of diseases responsible for admission in the future, all ICD-10 codes were translated to the ICD-11 codes (version 05/2021) using WHO mapping tables with the correspondence between ICD-10 and ICD-11 codes and link the ICD-11 codes with their corresponding associated descriptive labels. Two independent investigators (VQD and BTKL) manually reviewed any non-unique code or discrepancies in mapping between ICD-10 and ICD-11 which were eventually resolved by the consensus of 2 reviewers with the reference to the majority of code assignments by treating doctors.

The outcomes from the hospitalization were defined as death in patients who died in the hospital or was going home to die imminently after hospital discharge (palliative discharge). Palliative discharge is a common practice in Vietnam when a patient is in a moribund state and expresses a wish to die at home. Deterioration was defined as any worsening of clinical conditions by the treating doctor's judgment at the time of discharge.

Statistical Analysis

For the analysis of pattern of admission, we included all episodes of admission. Consequently, one patient might have multiple episodes of admission to CCUs during a year. For each episode, the primary diagnosis which was defined as the main reason for CCU

For peer review only - http://bmjopen.bmj.com/site/about/guidelines.xhtml

admission was counted. The primary diagnosis for that episode may be the same or different from the previous episode of admission. Individual patients were identified within the datasets by matching on full name, gender, date of birth and insurance numbers when they were available). The in-hospital mortality was calculated by dividing the number of deaths by the total number of individual patients.

Years of life lost (YLLs) is a measure of premature mortality which is calculated from the number of deaths at each age multiplied by a standard life expectancy for the age at which death occurs [9]. Vietnam's standard life expectancy at birth in 2019 for both sexes were 73.7 years old [10] and to compare with other studies, we considered premature death were deaths under 75 years old. We also calculated the average YLL by dividing total of YLL by the number of premature death and the crude YLL rate by dividing total of YLL by the total number of patients aged under 75 years (expressed per 1000 persons).

We presented categorical variables as frequencies and percentages, while continuous variables as medians and interquartile ranges [IQR]. We used the chi-square test or Fisher's exact test to compare two binomial proportions. Statistical analyses were performed using SPSS version 27 (IBM, Armonk, NY, USA). Significant differences were considered if two-sided P values were < 0.05.

Patient and public involvement

Patients and the public were not involved in the design, recruitment or conduct of the study.

Results

Between 1 January 2018 and 31 December 2018, there were 44,013 admission episodes to CCUs from 34 hospitals in 5 provinces (namely Hanoi, Thai Nguyen, Ha Nam, Kon Tum and Can Tho), corresponding to 38534 unique patients. There were 3,647 (9.5%) patients with two or more admission in the study year (Figure 1). The median age was 53 years (interquartile range [IQR]) 25-70) and included 53.4% male. The majority of CCU admissions in this study (31,569, 71.7%) were at the district hospitals. The median length of stay was 5 (IQR 2-8) days. A total of 1,764/36,518 admissions (4.8%) had a length of stay of more than

For peer review only - http://bmjopen.bmj.com/site/about/guidelines.xhtml

14 days. The hospitalization outcome was available for 28,311/38,534 (73.5%) patients. Overall survival to hospital discharge was 94.1% (26,630/28,311).

Admission diagnosis in critical care units

Of 44,013 episodes of admission to CCUs, the most common diagnosis were respiratory diseases (12,255 episodes, 27.8%) in which, 38.7% was certain lower respiratory tract diseases (blockL1-CA2) and 34.4% was lung infections (blockL1-CA4), followed by unclassified symptoms, signs or clinical findings (5,712 episodes or 13.0%) (51.8% was of digestive system or abdomen, blockL1-MD8), circulatory system diseases (5,380 episodes, 12.2%) (51,2% was hypertensive diseases, blockL1-BA0), diseases of the nervous system (5,019 episodes, 11.4%) (85.4% was cerebrovascular diseases, blockL1-8B0) and injury, poisoning or consequences of external causes (4,186 episodes or 9.5%) (39.7% was harmful effect off substances, blockL1-NE6). The percentage of diagnosis in episodes of CCU admission varied by age groups (Figure 2). The number of admissions peaked in children from 5 years old and younger and was lowest in the 5-10 year age group (Figure 3). The respiratory disease was the leading cause of admission in the under 10 year age group (64% of admissions) and age groups of 61 years and older (29% of admissions) (Figure 2)

The number of admission in all study hospitals fluctuated across months but mostly driven by the admission by respiratory diseases (Supplementary Figure 1). The proportions of admission to CCUs were significantly higher in provincial hospitals than in district hospital in regard to diseases of the nervous system (18.7% vs 8.5%, $p<0.001$), certain infectious or parasitic diseases (8.9% vs 3.4%, $p<0.001$), injury/poisoning (10.3% vs 9.2%, $p=0.00$), endocrine, nutritional or metabolic diseases (3.6% vs 2.2%, $p<0.001$) and neoplasms (2% vs 0.7%, $p<0.001$) (Figure 4**Error! Reference source not found.**).

Outcome of hospitalization

The median length of stay for all admissions was 5 days (IQR 2-8). Among 28,311 patients with available outcome data and, 1,681/28,311 (5.9%) died. The median age of death was 68 years (IQR 47-86) and 63.3% of death were in men. Men had significantly higher in-hospital mortality rate than women (3.8% vs. 2.6%, $p<0.001$). The median duration of hospital stay for the survivors and death cases was 4 (IQR:2-8) and 3 (IQR: 2–7), respectively. More than three quarters of deaths (1,290/1,681 or 76.7%) occurred in provincial hospitals. The all-cause in-hospital mortality rates by age groups and mortality

For peer review only - http://bmjopen.bmj.com/site/about/guidelines.xhtml

176 rate for selected groups of diagnosis are presented in Figure 3 and Supplementary Table 1.
177 The most common causes of death by ICD-11 block codes and chapter codes are showed in
178 Table 1 and Supplementary Table 2, respectively.

For peer review only

For peer review only - http://bmjopen.bmj.com/site/about/guidelines.xhtml

Table 1. The top 20 causes of death in terms of years of life lost (YLL) and numbers of deaths by ICD-11 block level 1 codes

Diagnosis	Number and proportion of death cases	In-hospital mortality (n,%)	Total YLL75	Proportion of YLLs75	YLL75 per 1000 patients	Length of stay to death (median, IQR)
Cerebrovascular diseases	303 (18.0%)	8.7%	3211000	12.3%	1380	3 (2-6)
Miscellaneous	259 (15.4%)	2.3%	5686000	38.2%	614	2 (1-6)
Lung infections	201 (12.0%)	7.2%	2875000	9.5%	1440	7 (3-15)
Sepsis	149 (8.9%)	36.8%	1991000	2.6%	8127	4 (2-11)
Harmful effects of substances	87 (5.2%)	6.1%	3534000	5.0%	2544	1 (1-2)
Respiratory failure	79 (4.7%)	18.1%	365000	1.6%	1714	9 (5-15)
General symptoms, signs or clinical findings	67 (4.0%)	13.5%	1507000	2.2%	3712	2 (1-4.5)
Injuries to the head	66 (3.9%)	15.6%	1140000	1.6%	3167	2 (2-4)
Certain lower respiratory tract diseases	63 (3.7%)	2.1%	443000	10.2%	217	4 (2-10)
Symptoms, signs or clinical findings of the digestive system or abdomen	61 (3.6%)	5.5%	994000	4.0%	1062	3 (2-6)
Malignant neoplasms, except primary neoplasms of lymphoid, haematopoietic, central nervous system or related	50 (3.0%)	18.0%	587000	1.0%	3073	6 (3-9)
Symptoms, signs or clinical findings of the circulatory system	50 (3.0%)	31.8%	1060000	0.7%	8217	2 (1-6)
Heart failure	39 (2.3%)	8.4%	280000	1.6%	1466	3 (1-5)
Ischaemic heart diseases	37 (2.2%)	5.2%	78000	2.5%	158	3 (1-5)
Diseases of liver	36 (2.1%)	11.2%	549000	1.1%	1861	2.5 (1.5-7.5)
Injuries involving multiple body regions	29 (1.7%)	28.2%	439000	0.4%	6362	2 (1-6)
Symptoms, signs or clinical findings of the respiratory system	28 (1.7%)	8.4%	706000	1.1%	3017	2 (1-4)
Mental or behavioural symptoms, signs or clinical findings	22 (1.3%)	13.3%	363000	0.8%	2771	2 (1-2)
Allergic or hypersensitivity conditions	20 (1.2%)	3.3%	890000	2.2%	1537	2 (1-2)
Infection, unspecified	19 (1.1%)	31.7%	741000	0.2%	14529	2 (2-4)
Epilepsy or seizures	16 (1.0%)	5.0%	696000	1.1%	2275	1.5 (1-2)
Total	1681 (100.0%)	5.9%	28135000	100.0%	1287	3 (2-7)

For peer review only

For peer review only - http://bmjopen.bmj.com/site/about/guidelines.xhtml

The in-hospital mortality in patients over 75 years was 662/6,140 (10.8%). In total, 28,135,000 years were lost among patient admitted to the study CCUs. This yielded an overal YLL under age of 75 years of 1,287 per 1,000 people. Table 1 presents the top 10 causes of death by ICD-11 block codes. Cerebrovascular diseases rank the first in the number of deaths (303/1,681 or 18%) and the eleven when measuring in in-hospital mortality (8.7%) while sepsis ranked as the first specified cause of death in terms of in-hospital mortality (36.8%) and ranked fourth in term of death counts.

Discussion

This study presents for the first time, data to quantify the burden of diseases in CCUs, for the most common reasons of admission and the in-hospital mortality of patients admitted to CCUs in Vietnam. Few studies on admission to the CCUs in lower-middle income countries have been reported earlier.

Burden of diseases in Vietnam has changed significantly in the past 20 years [4]. The communicable diseases burden (measured in disability-adjusted life years) decreased rapidly from 37% in 1990 to 14% in 2017. However, in the critical care settings, our study shows that the infectious diseases remain a major emergency medical condition and are the prime cause of morbidity and mortality in terms of number of admission and in terms of YLL.

Our findings on the difference distribution of diseases in CCUs in provincial and district hospitals reflected the variation of healthcare services and capacity of clinical management. In 2019, we conducted a survey on the capacity and use of diagnostics and treatment for patients with severe acute respiratory infections in 48 hospitals in Vietnam (including 32 hospitals in this study) at the same time as this study. Significant differences were observed in the district hospitals when compared to provincial hospitals in regards to availability of the microbiological diagnostics (9.4% vs 81.2%, $p<0.001$), inflammatory markers of C-reactive protein (31.2% vs 54.2%, $p<0.001$) and procalcitonine (3.1% vs 25%, $p<0.001$) and mechanical ventilation (40.6% vs 60.4%, $p<0.001$)[11]. Provincial hospitals in Vietnam are the secondary hospitals which do not only treat referred patients from lower level of care (district hospitals or commune centres) but also admit patients from the community. Additionally, patient can directly self-refer to higher level of hospitals which resulting in

For peer review only - http://bmjopen.bmj.com/site/about/guidelines.xhtml

under-utilization of district hospitals and over crowding in provincial hospitals, especially for given specialities which requires higher levels of skills and capacity.

Our findings are partly aligned with previous reports in all cause mortality in both sexes and all ages showing that cardiovascular (ischaemic heart disease, stroke), respiratory diseases and injuries were the significant challenge. WHO's report in 2019 showed that ischaemic heart disease, stroke, neonatal conditions, chronic obstructive pulmonary disease (COPD) and lower respiratory infections were the top 5 causes of death in term of death counts in LMICs [12]. At the global level in 2019, the top three diseases with highest burden (measuring in DALY) were neonatal disorder (32.9% of global DALYs), lower respiratory infections (11% of global DALYs) and diarrhoeal diseases (9.3% of global DALYs) in children younger than 10 years and were ischemic heart diseases (16.2% of global DALY), stroke (13% of global DALY) and COPD (8.5% of global DALY) in patients from 75 years and older [13]. In Vietnam, stroke (164.8 deaths per 100,000), ischaemic heart disease (95.3 deaths per 100,000), COPD (37.2 deaths per 100,000) and diabetes mellitus (35.1 deaths per 100,000) and road injuries (30.5 deaths per100,000) were in the top-five rankings for total number of death in 2019 [14]. Sepsis and harmful effects of substances were not listed in the top 5 cause of death in the general population, but we found that these were important conditions in emergency care settings.

The overall mortality in our study were higher than previous report. In a systematic review of data from 65 emergency departments in 59 LMICs countries between 2013 and 2014, the median mortality was 1.8% (IQR 0.5-5.1%), highest in sub-Saharan Africa (median 3.4%, IQR: 0.5–6.3%) and lowest in South Asia, East Asia & Pacific (0.3, IQR 0.2–0.8%) [15].

In our study, patients aged 75 years or older presented 18.9% of all admission at CCUs despite the fact that they comprised 3.2% of general population [16]. The percentage of population older than 75 years in Vietnam is expected to increase to 4.2% by 2030 and 6.6% by 2040 [16]. This will increase the demand for critical care resources and requires further evaluation to guide healthcare decision-making and changes in organizational models in CCU and delivery of critical care services during a pandemic.

The design of multicentre study, large population and data collection for the whole year of 2018 are the strengths of our study. The major limitations related to the diagnosis

For peer review only - http://bmjopen.bmj.com/site/about/guidelines.xhtml

description and diagnostic coding by doctors in study hospitals. Data quality of diagnosis may vary between hospitals due to the accurate clinical diagnosis of disease depends on doctor's knowledge and skills, available laboratory investigations and diagnostic imaging procedures. However, there are differences in the capacity of clinical management and use of diagnostics between provincial and district hospitals in the country [11]. In Vietnam, treating doctors are responsible for assigning the ICD diagnostic code to each patient discharged from the hospital. The complex nature of ICD coding, patient’s condition in critical care settings and the lack of coding experience pose further challenges for the accuracy and reliability of the diagnosis given across hospitals. Another limitation was the missing information on outcome for 26.5% (10,223/38543) of patients because these were not actually entered into the electronic hospital management system. This missing data can cause bias in the estimation of in-hospital mortality.

Currently, there is limited cost-effectiveness analysis performed for critical care services in Vietnam. The results of this study in regards to the a variety of diagnoses and outcomes of admission will direct the future studies on the establishment of CCU registry, the effectiveness of CCU treatments and estimates of the total cost to save a life-year. This information will be helpful to develop policies and strategies to make better-informed decisions in resource-constrained settings in the face of an aging population in Vietnam.

Conclusion

Our study indicates a variation of needs of emergency care by age groups and levels of care among patients admitted to CCUs in provincial and district hospitals in Vietnam. These findings support the need to provide multidisciplinary team training for clinicians in CCUs and the future studies should further assess the emergency and critical care capacity needs, ability of the healthcare setting to provide the emergency services and the quality of care (including minimal set of indicators) and gaps to mobilize the resources required for live saving during a pandemic.

Data availability statement

Data are available upon reasonable request.

For peer review only - http://bmjopen.bmj.com/site/about/guidelines.xhtml

Ethics statements

Patient consent for publication

Not applicable

Ethics approval

This study was approved by the institutional review board (IRB) in the Hanoi Medical University (59/GCN-DDNCYSH-DHYHN).

Funding statement

This work was supported by Viet Nam - WHO Country Office. The funder of the study had no role in study design, data collection, data analysis, data interpretation, or writing of the report.

Competing interests

We declare no competing interest.

Contributors

VQD conceived and designed the study. VQD analysed the data and wrote the first draft of the report. VQD, BTKL and KBG involved to the acquisition and interpretation of data. All authors contributed to and approved the final report.

Acknowledgments

We are very grateful to the patients and staff at the participating hospitals in Ha Noi, Thai Nguyen, Ha Nam, Kon Tum and Can Tho. We would like to thank Drs. Satoko Otsu and Vu Quang Hieu (World Health Organization, Vietnam Country Office) for their tireless support and contribution to strengthening the capacity for clinicans in Vietnam and thank Dr. Nguyen The Hung (National Hospital for Tropical Diseases) for his initial support in the implementation of this study.

REFERENCES

1. Razzak J, Usmani MF, Bhutta ZA. Global, regional and national burden of emergency medical diseases using specific emergency disease indicators: analysis of the 2015 Global Burden of Disease Study. *BMJ Glob Health* 2019;4(2):e000733. doi: 10.1136/bmjgh-2018-000733
2. World Health Assembly. Emergency care systems for universal health coverage: ensuring timely care for the acutely ill and injured. Geneva: World Health Organization, 2019.

For peer review only - http://bmjopen.bmj.com/site/about/guidelines.xhtml

3. Executive Board. International Classification of Diseases: International Statistical Classification of Diseases and Related Health Problems: update on the eleventh revision: report by the Director-General. Geneva: World Health Organization, 2018.
4. Elena Glinskaya, Annette De Kleine Feige, Vu Thi Lan Hoang, et al. Vietnam - Adapting to an Aging Society Washington, D.C: World Bank Group, 2021.
5. Nguyen NTT, Dien TM, Schindler C, et al. Childhood hospitalisation and related deaths in Hanoi, Vietnam: a tertiary hospital database analysis from 2007 to 2014. *BMJ open* 2017;7(7):e015260. doi: 10.1136/bmjopen-2016-015260
6. Hong TT, Phuong Hoa N, Walker SM, et al. Completeness and reliability of mortality data in Viet Nam: Implications for the national routine health management information system. *PloS one* 2018;13(1):e0190755. doi: 10.1371/journal.pone.0190755
7. Walton M, Harrison R, Chevalier A, et al. Improving hospital death certification in Vietnam: results of a pilot study of injury-related fatalities. *Inj Prev* 2018;24(5):324-31. doi: 10.1136/injuryprev-2017-042341
8. Murthy S, Leligdowicz A, Adhikari NK. Intensive care unit capacity in low-income countries: a systematic review. *PloS one* 2015;10(1):e0116949. doi: 10.1371/journal.pone.0116949
9. World Health Organization. WHO methods and data sources for global burden of disease estimates 2000-2019. In: Analytics DoDa, Division of Data AaDfI, eds. Geneva: World Health Organization,, 2020.
10. World Health Organization. Life expectancy and Healthy life expectancy. Data by country 2020 [updated 4th December 2021; cited 2022 6th January]. Available from: https://apps.who.int/gho/data/node.main.688 accessed 6th January 2022.
11. Dat VQ, Hung NT, Giang BK, et al. Capacity and use of diagnostics and treatment for patients with severe acute respiratory infections in the pre-COVID-19 era in district and provincial hospitals in Viet Nam. *Western Pac Surveill Response J* 2021;12(4):1-9. doi: doi.org/10.5365/wpsar.2021.12.4.835
12. World Health Organization. The top 10 causes of death 2019 [updated 9th December 2020; cited 2021 11th December]. Available from: https://www.who.int/news-room/fact-sheets/detail/the-top-10-causes-of-death accessed 11th December 2021.
13. GBD 2019 Diseases and Injuries Collaborators. Global burden of 369 diseases and injuries in 204 countries and territories, 1990-2019: a systematic analysis for the Global Burden of Disease Study 2019. *Lancet* 2020;396(10258):1204-22. doi: 10.1016/S0140-6736(20)30925-9
14. World Health Organization. Global Health Estimates 2020: Deaths by Cause, Age, Sex, by Country and by Region, 2000-2019 2020 [cited 2021 13th December]. Available from: https://www.who.int/data/gho/data/themes/mortality-and-global-health-estimates/ghe-leading-causes-of-death accessed 13th December 2021.
15. Obermeyer Z, Abujaber S, Makar M, et al. Emergency care in 59 low- and middle-income countries: a systematic review. *Bull World Health Organ* 2015;93(8):577-86G. doi: 10.2471/BLT.14.148338
16. General Statistic Office, United Nations Population Fund. Vietnam population projection for the period 2019-2069. In: Office GS, ed. Hanoi, 2020.

Figure Legend

Figure 1. Flowchart of CCU admission and outcome.

Figure 2. Diangosis on admission to CCU by age groups.

Figure 3. Number of admission and In-hospital mortality by age groups

Figure 4. Cause of admission to CCUs by level of care.

For peer review only - http://bmjopen.bmj.com/site/about/guidelines.xhtml

Supplementary materials

Supplementary Figure 1. Pattern of admissions over months.

Supplementary Table 1. Mortality and years of life lost (YLL) grouped by ICD-11 chapter codes

Supplementary Table 2. The number of deaths by ICD-11 4-character codes

For peer review only

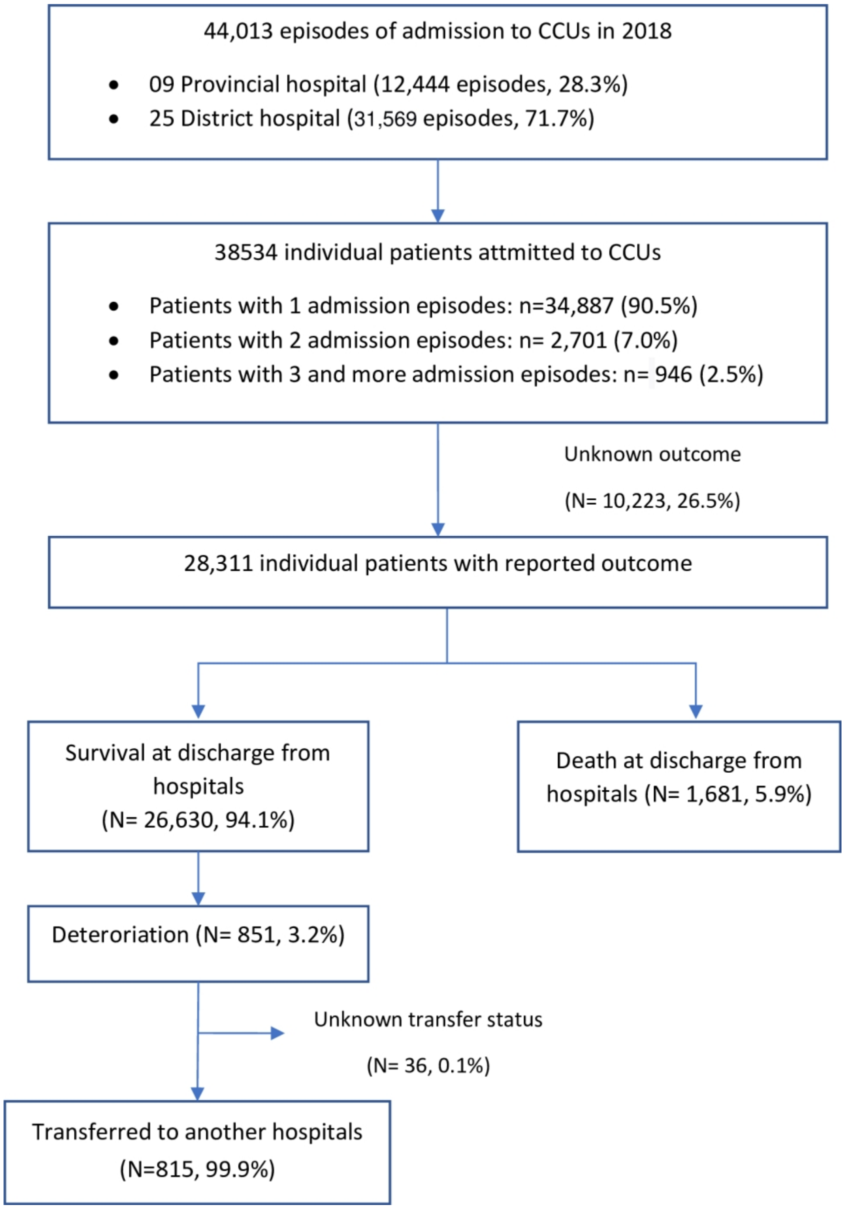

Figure 1. Flowchart of CCU admission and outcome.

129x185mm (300 x 300 DPI)

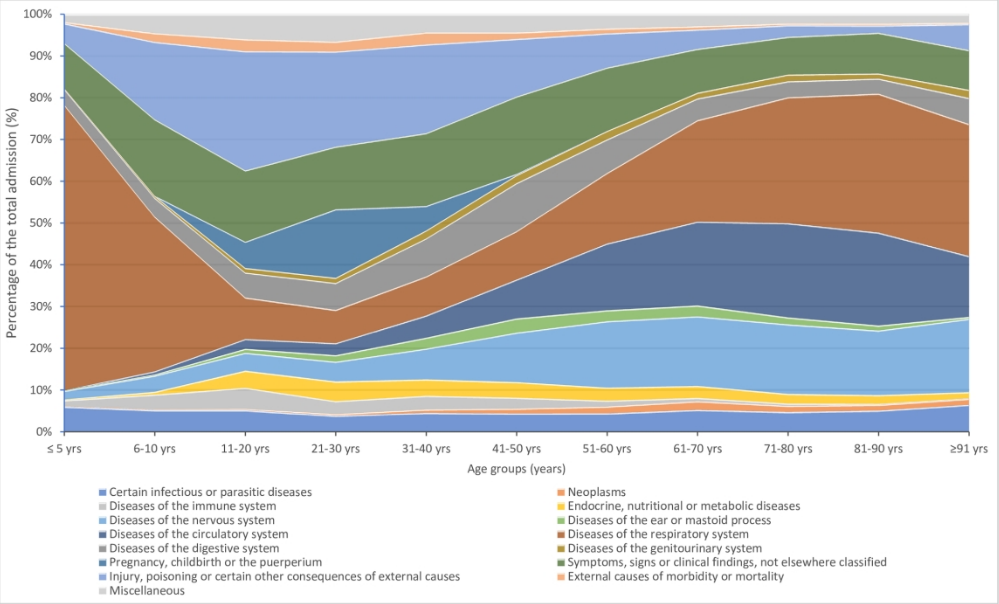

Figure 2. Diangosis on admission to CCU by age groups.

190x115mm (300 x 300 DPI)

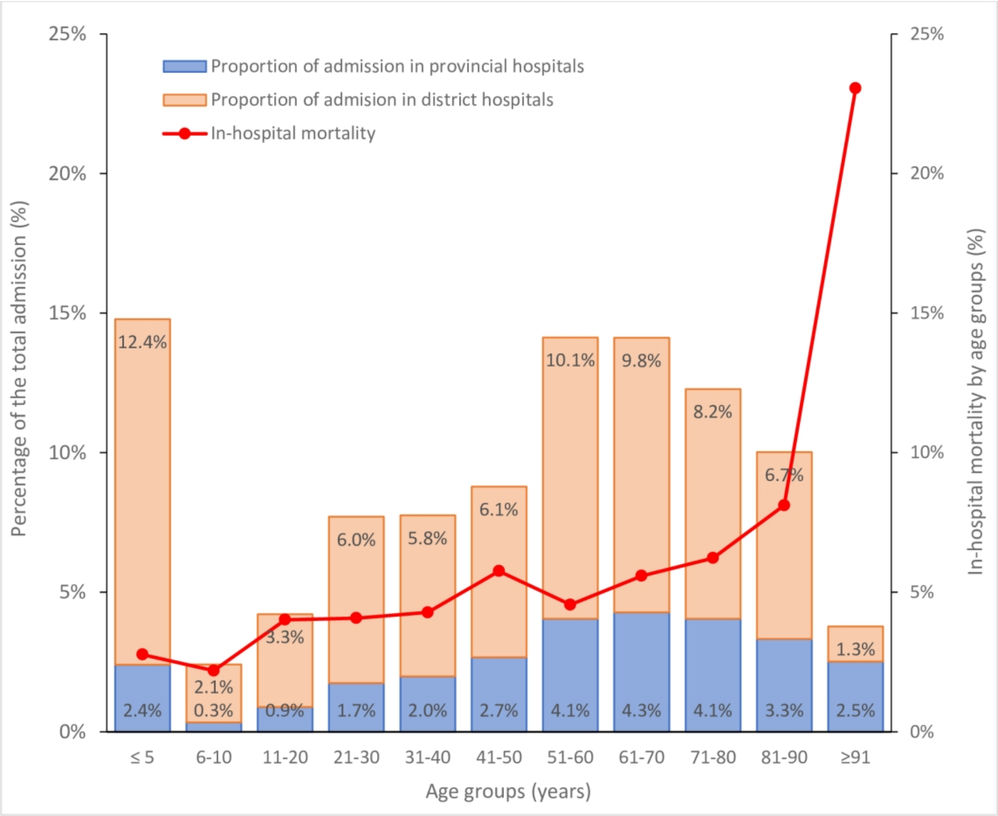

Figure 3. Number of admission and In-hospital mortality by age groups

159x129mm (300 x 300 DPI)

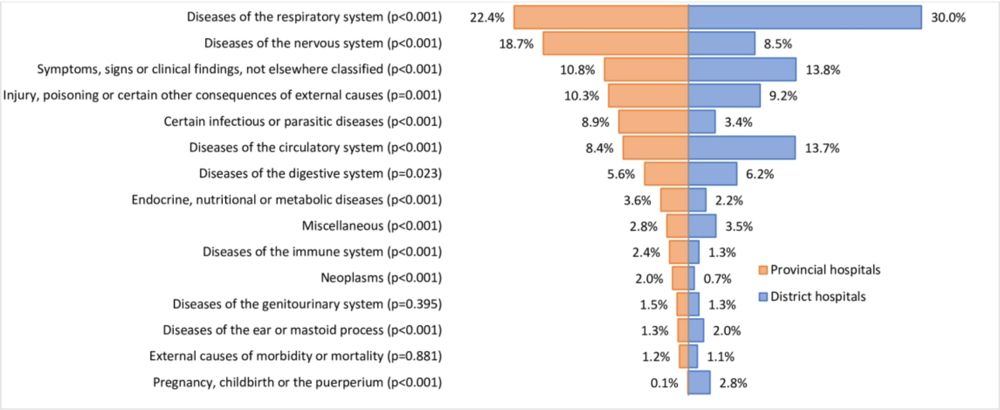

Figure 4. Cause of admission to CCUs by level of care.

190x77mm (300 x 300 DPI)

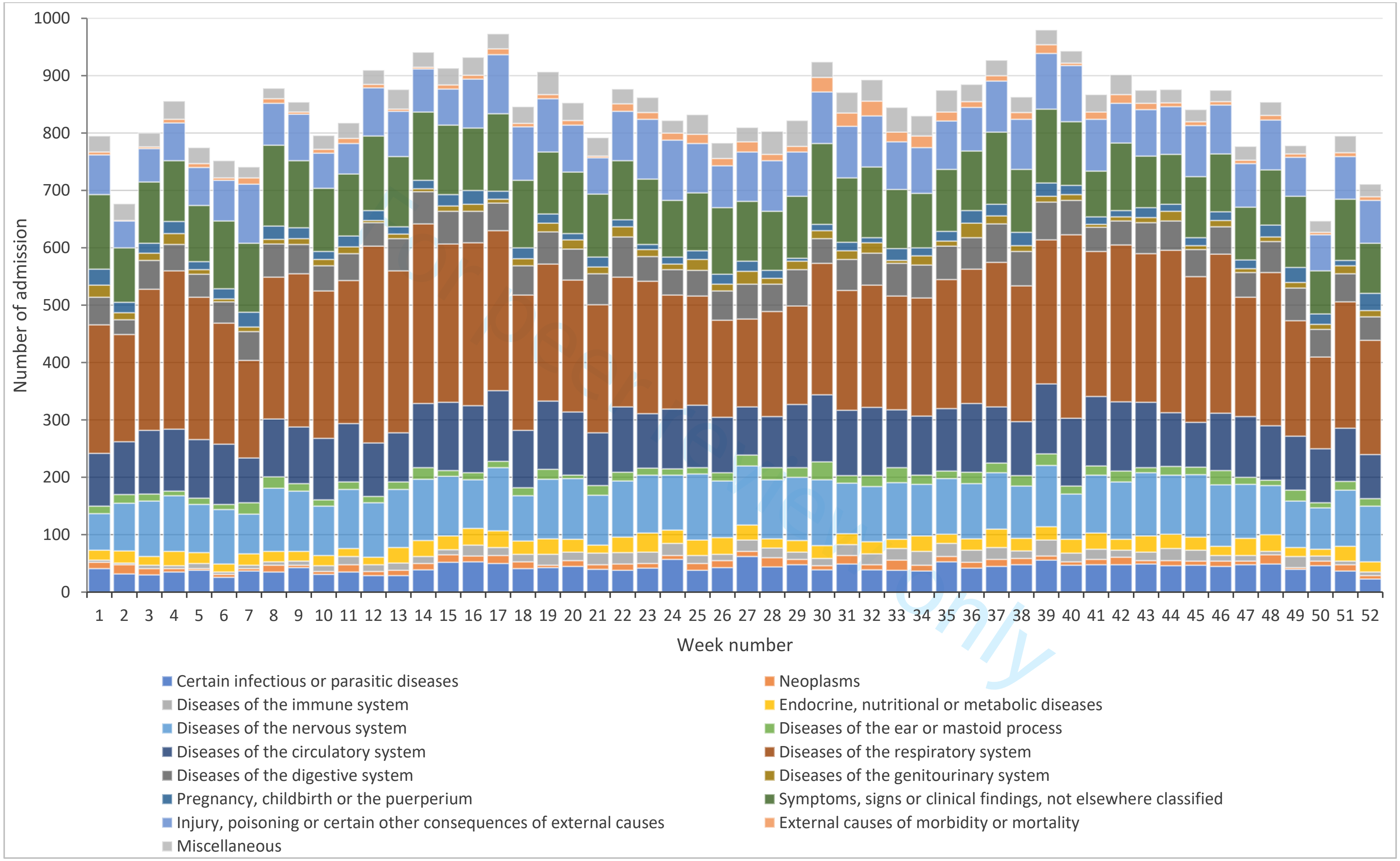

Supplementary Figure 1. Pattern of admissions over months.

Supplementary Table 1. Mortality and years of life lost (YLL) grouped by ICD-11 chapter codes

Diagnosis	Number and proportion of death cases	In-hospital mortality (n,%)	Total YLL75	Proportion of YLLs75	YLL75 per 1000 patients	Length of stay to death (median, IQR)
Diseases of the respiratory system	367 (21.8%)	4.8%	4506000	25.9%	813	6 (3-13)
Diseases of the nervous system	324 (19.3%)	8.1%	3994000	14.0%	1432	2 (2-6)
Symptoms, signs or clinical findings, not elsewhere classified	241 (14.3%)	9.4%	5141000	9.8%	2476	2 (1-4)
Injury, poisoning or certain other consequences of external causes	210 (12.5%)	8.4%	5846000	8.8%	2557	2 (1-2)
Certain infectious or parasitic diseases	188 (11.2%)	14.0%	3218000	5.8%	3056	4 (2-10)
Diseases of the circulatory system	112 (6.7%)	2.8%	817000	13.4%	314	3 (1-5)
Diseases of the digestive system	83 (4.9%)	3.9%	1221000	7.1%	683	3 (2-8)
Neoplasms	59 (3.5%)	17.3%	809000	1.3%	3385	5 (2-9)
Miscellaneous	31 (1.8%)	3.5%	963000	3.1%	1259	2 (1-5)
Endocrine, nutritional or metabolic diseases	22 (1.3%)	2.4%	217000	3.3%	276	2 (1-3)
Diseases of the immune system	20 (1.2%)	3.3%	890000	2.2%	1532	2 (1-2)
Diseases of the genitourinary system	13 (0.8%)	3.0%	168000	1.5%	487	5 (2-11)
External causes of morbidity or mortality	8 (0.5%)	1.8%	223000	1.6%	556	3.5 (1.5-4.5)
Diseases of the ear or mastoid process	2 (0.1%)	0.4%	87000	1.8%	192	1 (1-1)
Pregnancy, childbirth or the puerperium	1 (0.1%)	0.7%	35000	0.5%	241	1 (1-1)
Total	1681 (100.0%)	5.9%	28135000	100.0%	1287	3 (2-7)

For peer review only

Supplementary Table 2. The number of deaths by ICD 4-character codes

	Causes of death	N (%)
**CHAPTER 01 Certain infectious or parasitic diseases**	Viral intestinal infections, unspecified	1 (0.06%)
	Gastroenteritis or colitis without specification of infectious agent	1 (0.06%)
	Tuberculosis of the respiratory system	2 (0.12%)
	Bacterial cellulitis, erysipelas and lymphangitis	1 (0.06%)
	Other specified pyogenic bacterial infection of skin and subcutaneous tissue	1 (0.06%)
	Tetanus	1 (0.06%)
	Bacterial infection of unspecified site	1 (0.06%)
	Viral encephalitis not elsewhere classified	2 (0.12%)
	Viral meningitis not elsewhere classified	1 (0.06%)
	Infectious abscess of the central nervous system	1 (0.06%)
	Dengue fever, unspecified	1 (0.06%)
	Acute viral hepatitis	4 (0.24%)
	Viral hepatitis, unspecified	2 (0.12%)
	Phaeohyphomycosis	1 (0.06%)
	Sepsis without septic shock	23 (1.37%)
	Sepsis with septic shock	126 (7.50%)
	Infection, unspecified	19 (1.13%)
**CHAPTER 02 Neoplasms**	Primary neoplasms of brain	6 (0.36%)
	Malignant haematopoietic neoplasms without further specification	1 (0.06%)
	Malignant neoplasms of other or ill-defined sites in the lip, oral cavity or pharynx	2 (0.12%)
	Malignant neoplasms of oesophagus	5 (0.30%)
	Malignant neoplasms of stomach	2 (0.12%)
	Malignant neoplasms of colon	5 (0.30%)
	Malignant neoplasm of pancreas	1 (0.06%)
	Malignant neoplasms of liver or intrahepatic bile ducts	11 (0.65%)
	Malignant neoplasms of gallbladder	1 (0.06%)
	Malignant neoplasms of bronchus or lung	10 (0.59%)
	Malignant neoplasms of thymus	1 (0.06%)
	Malignant neoplasms of heart, mediastinum or non-mesothelioma of pleura	2 (0.12%)
	Malignant neoplasms of breast, unspecified	3 (0.18%)
	Malignant neoplasms of prostate	1 (0.06%)
	Malignant neoplasms of bladder	1 (0.06%)
	Malignant neoplasms of thyroid gland	1 (0.06%)
	Malignant neoplasm metastasis in lung	4 (0.24%)
	Carcinoma in situ of bladder	1 (0.06%)
	Benign osteogenic tumours	1 (0.06%)
**CHAPTER 03 Diseases of the blood or blood-forming organs**	Anaemia due to chronic disease	1 (0.06%)
	Anaemias or other erythrocyte disorders, unspecified	8 (0.48%)
	Anaphylaxis	9 (0.54%)

For peer review only

	Causes of death	N (%)
**CHAPTER 04 Diseases of the immune system**	Allergic or hypersensitivity conditions of unspecified type	11 (0.65%)
**CHAPTER 05 Endocrine, nutritional or metabolic diseases**	Type 1 diabetes mellitus	3 (0.18%)
	Type 2 diabetes mellitus	3 (0.18%)
	Hypoglycaemia without associated diabetes	5 (0.30%)
	Increased secretion of glucagon	1 (0.06%)
	Protein deficiency	1 (0.06%)
	Hyperosmolality or hypernatraemia	1 (0.06%)
	Acidosis	1 (0.06%)
	Hyperkalaemia	1 (0.06%)
	Hypokalaemia	1 (0.06%)
	Disorders of fluid, electrolyte or acid-base balance, unspecified	5 (0.30%)
**CHAPTER 06 Mental, behavioural or neurodevelopmental disorders**	Developmental learning disorder	1 (0.06%)
	Dissociative disorders, unspecified	4 (0.24%)
	Disorders due to use of dissociative drugs including ketamine and phencyclidine [PCP]	1 (0.06%)
	Delirium	1 (0.06%)
	Mental, behavioural or neurodevelopmental disorders, unspecified	2 (0.12%)
**CHAPTER 08 Diseases of the nervous system**	Seizure due to acute causes	2 (0.12%)
	Types of seizures	11 (0.65%)
	Epilepsy or seizures, unspecified	3 (0.18%)
	Intracerebral haemorrhage	133 (7.91%)
	Subarachnoid haemorrhage	3 (0.18%)
	Intracranial haemorrhage, unspecified	32 (1.90%)
	Cerebral ischaemic stroke	72 (4.28%)
	Stroke not known if ischaemic or haemorrhagic	61 (3.63%)
	Late effects of cerebrovascular disease	2 (0.12%)
	Myelitis	1 (0.06%)
	Motor neuron disease	1 (0.06%)
	Hereditary sensory or autonomic neuropathy	1 (0.06%)
	Increased intracranial pressure	1 (0.06%)
	Disorders of the meninges excluding infection	1 (0.06%)
**CHAPTER 10 Diseases of the ear or mastoid process**	Disorders of vestibular function	2 (0.12%)
**CHAPTER 11 Diseases of the circulatory system**	Essential hypertension	8 (0.48%)
	Hypertensive heart disease	1 (0.06%)
	Hypotension, unspecified	5 (0.30%)
	Angina pectoris	1 (0.06%)
	Acute myocardial infarction	36 (2.14%)
	Pulmonary thromboembolism	1 (0.06%)
	Pulmonary hypertension	1 (0.06%)
	Pulmonary heart disease or diseases of pulmonary circulation, unspecified	5 (0.30%)
	Pericardial effusion	1 (0.06%)
	Mitral valve stenosis with insufficiency	1 (0.06%)

For peer review only

	Causes of death	N (%)
	Myocarditis	1 (0.06%)
	Conduction disorders	1 (0.06%)
	Ventricular tachyarrhythmia	2 (0.12%)
	Cardiac arrhythmia, unspecified	5 (0.30%)
	Congestive heart failure	3 (0.18%)
	Heart failure, unspecified	36 (2.14%)
	Acute arterial occlusion	1 (0.06%)
	Aortic aneurysm or dissection	1 (0.06%)
	Diseases of arteries or arterioles, unspecified	1 (0.06%)
	Deep vein thrombosis	1 (0.06%)
**CHAPTER 12 Diseases of the respiratory system**	Acute tonsillitis	1 (0.06%)
	Acute laryngitis or tracheitis	1 (0.06%)
	Acute upper respiratory infections of multiple and unspecified sites	1 (0.06%)
	Bronchitis	2 (0.12%)
	Chronic obstructive pulmonary disease	52 (3.09%)
	Asthma	8 (0.48%)
	Bronchiectasis	1 (0.06%)
	Pneumonia	198 (11.78%)
	Abscess of lung or mediastinum	2 (0.12%)
	Lung infections, unspecified	1 (0.06%)
	Pneumonitis due to solids and liquids	1 (0.06%)
	Respiratory conditions due to inhalation of chemicals, gases, fumes or vapours	3 (0.18%)
	Acute respiratory distress syndrome	2 (0.12%)
	Pulmonary oedema	11 (0.65%)
	Pneumothorax	2 (0.12%)
	Haemothorax	1 (0.06%)
	Pleural effusion	1 (0.06%)
	Respiratory failure	79 (4.70%)
**CHAPTER 13 Diseases of the digestive system**	Gastro-oesophageal reflux disease	1 (0.06%)
	Vascular disorders of the oesophagus	3 (0.18%)
	Gastritis	4 (0.24%)
	Gastric ulcer	5 (0.30%)
	Obstruction of small intestine	7 (0.42%)
	Appendicitis	3 (0.18%)
	Obstruction of large intestine	1 (0.06%)
	Polyp of large intestine	1 (0.06%)
	Infectious liver disease	1 (0.06%)
	Acute or subacute hepatic failure	2 (0.12%)
	Hepatic fibrosis or cirrhosis	15 (0.89%)
	Autoimmune liver disease	1 (0.06%)
	Certain specified diseases of liver	17 (1.01%)
	Cholelithiasis	2 (0.12%)
	Cholangitis	2 (0.12%)
	Acute pancreatitis	8 (0.48%)
	Peritonitis	6 (0.36%)
	Inguinal hernia	1 (0.06%)
	Indeterminate colitis	1 (0.06%)

For peer review only

	Causes of death	N (%)
	Diseases of the digestive system, unspecified	2 (0.12%)
**CHAPTER 14 Diseases of the skin**	Lichen planus	1 (0.06%)
	Skin disease of unspecified nature	1 (0.06%)
**CHAPTER 16 Diseases of the genitourinary system**	Pyonephrosis	1 (0.06%)
	Acute kidney failure	1 (0.06%)
	Chronic kidney disease	7 (0.42%)
	Kidney failure, unspecified	2 (0.12%)
	Urinary tract infection, site not specified	2 (0.12%)
**CHAPTER 18 Pregnancy, childbirth or the puerperium**	Complications following abortion, ectopic or molar pregnancy	1 (0.06%)
**CHAPTER 19 Certain conditions originating in the perinatal period**	Sepsis of foetus or newborn	1 (0.06%)
	Respiratory failure of newborn	1 (0.06%)
	Transitory disorders of carbohydrate metabolism specific to foetus or newborn	1 (0.06%)
	Certain specified transitory neonatal electrolyte or metabolic disturbances	1 (0.06%)
**CHAPTER 20 Developmental anomalies**	Congenital hydrocephalus	1 (0.06%)
	Congenital anomaly of a ventricle or the ventricular septum	1 (0.06%)
	Structural developmental anomalies of gallbladder, bile ducts or liver	1 (0.06%)
**CHAPTER 21 Symptoms, signs or clinical findings, not elsewhere classified**	Symptoms, signs or clinical findings involving consciousness	22 (1.31%)
	Abnormalities of heart beat	2 (0.12%)
	Cardiac arrest	48 (2.86%)
	Abnormalities of breathing	2 (0.12%)
	Haemorrhage from throat	1 (0.06%)
	Haemoptysis	2 (0.12%)
	Respiratory arrest	23 (1.37%)
	Abdominal or pelvic pain	4 (0.24%)
	Nausea or vomiting	1 (0.06%)
	Clinical manifestations of the digestive system	56 (3.33%)
	Cachexia	1 (0.06%)
	Fever of other or unknown origin	4 (0.24%)
	Haemorrhage, not elsewhere classified	1 (0.06%)
	Shock	60 (3.57%)
	Syncope and collapse	1 (0.06%)
	Other sudden death, cause unknown	1 (0.06%)
	Other ill-defined and unspecified causes of mortality	9 (0.54%)
	Multi organ failure	3 (0.18%)
**CHAPTER 22 Injury, poisoning or certain other consequences of external causes**	Injury of cranial nerves	1 (0.06%)
	Intracranial injury	65 (3.87%)
	Superficial injury of thorax	2 (0.12%)
	Injury of blood vessels at abdomen, lower back or pelvis level	1 (0.06%)
	Injury of intra-abdominal organs	5 (0.30%)
	Traumatic amputation of forearm	1 (0.06%)

For peer review only

	Causes of death	N (%)
	Fracture of femur	1 (0.06%)
	Superficial injury of ankle or foot	1 (0.06%)
	Crushing injuries involving multiple body regions	1 (0.06%)
	Unspecified multiple injuries	28 (1.67%)
	Burn of wrist or hand	1 (0.06%)
	Burns of external body surface, other specified site	1 (0.06%)
	Burns of multiple body regions	1 (0.06%)
	Harmful effects of drugs, medicaments or biological substances, not elsewhere classified	57 (3.39%)
	Harmful effects of or exposure to noxious substances, chiefly nonmedicinal as to source, not elsewhere classified	30 (1.78%)
	Effects of other specified external causes	3 (0.18%)
	Certain early complications of trauma, not elsewhere classified	11 (0.65%)
**CHAPTER 23 External causes of morbidity or mortality**	Unintentional exposure to or harmful effects of pesticides	1 (0.06%)
	Intentional self-harm by threat to breathing by suffocation from object covering mouth or nose	1 (0.06%)
	Intentional self-harm by and exposure to other and unspecified drug, medicament and biological substance	3 (0.18%)
	Intentional self-harm by exposure to or harmful effects of pesticides	1 (0.06%)
	Intentional self-harm by exposure to or harmful effects of other or unspecified substances chiefly nonmedicinal as to so	1 (0.06%)
	Exposure to or harmful effects of undetermined intent of other or unspecified drugs, medicaments or biological substance	1 (0.06%)
**CHAPTER 24 Factors influencing health status or contact with health services**	Surgical or postsurgical states, unspecified	3 (0.18%)
	Personal history of malignant neoplasm	1 (0.06%)

For peer review only

STROBE Statement—Checklist of items that should be included in reports of ***cross-sectional studies***

	Item No	Recommendation	Page No
**Title and abstract**	1	(*a*) Indicate the study's design with a commonly used term in the title or the abstract	1
		(*b*) Provide in the abstract an informative and balanced summary of what was done and what was found	2
**Introduction**			
Background/rationale	2	Explain the scientific background and rationale for the investigation being reported	4
Objectives	3	State specific objectives, including any prespecified hypotheses	5
**Methods**			
Study design	4	Present key elements of study design early in the paper	5
Setting	5	Describe the setting, locations, and relevant dates, including periods of recruitment, exposure, follow-up, and data collection	5
Participants	6	(*a*) Give the eligibility criteria, and the sources and methods of selection of participants	5
Variables	7	Clearly define all outcomes, exposures, predictors, potential confounders, and effect modifiers. Give diagnostic criteria, if applicable	5
Data sources/ measurement	8*	For each variable of interest, give sources of data and details of methods of assessment (measurement). Describe comparability of assessment methods if there is more than one group	*6*
Bias	9	Describe any efforts to address potential sources of bias	9
Study size	10	Explain how the study size was arrived at	5
Quantitative variables	11	Explain how quantitative variables were handled in the analyses. If applicable, describe which groupings were chosen and why	NA
Statistical methods	12	(*a*) Describe all statistical methods, including those used to control for confounding	6
		(*b*) Describe any methods used to examine subgroups and interactions	6
		(*c*) Explain how missing data were addressed	Fig 11
		(*d*) If applicable, describe analytical methods taking account of sampling strategy	NA
		(*e*) Describe any sensitivity analyses	NA
**Results**			
Participants	13*	(a) Report numbers of individuals at each stage of study—eg numbers potentially eligible, examined for eligibility, confirmed eligible, included in the study, completing follow-up, and analysed	6
		(b) Give reasons for non-participation at each stage	6
		(c) Consider use of a flow diagram	
Descriptive data	14*	(a) Give characteristics of study participants (eg demographic, clinical, social) and information on exposures and potential confounders	6
		(b) Indicate number of participants with missing data for each variable of interest	6
Outcome data	15*	Report numbers of outcome events or summary measures	6

For peer review only

Main results	16	(*a*) Give unadjusted estimates and, if applicable, confounder-adjusted estimates and their precision (eg, 95% confidence interval). Make clear which confounders were adjusted for and why they were included	7
		(*b*) Report category boundaries when continuous variables were categorized	NA
		(*c*) If relevant, consider translating estimates of relative risk into absolute risk for a meaningful time period	NA
Other analyses	17	Report other analyses done—eg analyses of subgroups and interactions, and sensitivity analyses	7
**Discussion**			
Key results	18	Summarise key results with reference to study objectives	8
Limitations	19	Discuss limitations of the study, taking into account sources of potential bias or imprecision. Discuss both direction and magnitude of any potential bias	9
Interpretation	20	Give a cautious overall interpretation of results considering objectives, limitations, multiplicity of analyses, results from similar studies, and other relevant evidence	9
Generalisability	21	Discuss the generalisability (external validity) of the study results	9
**Other information**			
Funding	22	Give the source of funding and the role of the funders for the present study and, if applicable, for the original study on which the present article is based	10

*Give information separately for exposed and unexposed groups.

Note: An Explanation and Elaboration article discusses each checklist item and gives methodological background and published examples of transparent reporting. The STROBE checklist is best used in conjunction with this article (freely available on the Web sites of PLoS Medicine at http://www.plosmedicine.org/, Annals of Internal Medicine at http://www.annals.org/, and Epidemiology at http://www.epidem.com/). Information on the STROBE Initiative is available at www.strobe-statement.org.

For peer review only

BMJ Open

Causes of admissions and in-hospital mortality among patients admitted to critical care units in primary and secondary hospitals in Vietnam in 2018: a multicentre retrospective study

Journal:	*BMJ Open*
Manuscript ID	bmjopen-2022-061638.R2
Article Type:	Original research
Date Submitted by the Author:	20-May-2022

Complete List of Authors:	Dat, Vu Quoc; Hanoi Medical University, Department of Infectious Diseases<br>Linh, Bui Thi Khanh; Hanoi Medical University, Department of Infectious Diseases<br>Kim, Giang; Hanoi Medical University

**Primary Subject Heading**:	Emergency medicine
Secondary Subject Heading:	Infectious diseases, Health policy, Epidemiology
Keywords:	Public health < INFECTIOUS DISEASES, Epidemiology < TROPICAL MEDICINE, Adult intensive & critical care < INTENSIVE & CRITICAL CARE, Epidemiology < INFECTIOUS DISEASES

SCHOLARONE™
Manuscripts

I, the Submitting Author has the right to grant and does grant on behalf of all authors of the Work (as defined in the below author licence), an exclusive licence and/or a non-exclusive licence for contributions from authors who are: i) UK Crown employees; ii) where BMJ has agreed a CC-BY licence shall apply, and/or iii) in accordance with the terms applicable for US Federal Government officers or employees acting as part of their official duties; on a worldwide, perpetual, irrevocable, royalty-free basis to BMJ Publishing Group Ltd ("BMJ") its licensees and where the relevant Journal is co-owned by BMJ to the co-owners of the Journal, to publish the Work in this journal and any other BMJ products and to exploit all rights, as set out in our licence.

The Submitting Author accepts and understands that any supply made under these terms is made by BMJ to the Submitting Author unless you are acting as an employee on behalf of your employer or a postgraduate student of an affiliated institution which is paying any applicable article publishing charge ("APC") for Open Access articles. Where the Submitting Author wishes to make the Work available on an Open Access basis (and intends to pay the relevant APC), the terms of reuse of such Open Access shall be governed by a Creative Commons licence – details of these licences and which Creative Commons licence will apply to this Work are set out in our licence referred to above.

Other than as permitted in any relevant BMJ Author's Self Archiving Policies, I confirm this Work has not been accepted for publication elsewhere, is not being considered for publication elsewhere and does not duplicate material already published. I confirm all authors consent to publication of this Work and authorise the granting of this licence.

For peer review only

Causes of admissions and in-hospital mortality among patients admitted to critical care units in primary and secondary hospitals in Vietnam in 2018: a multicentre retrospective study

Vu Quoc Dat[1], Bui Thi Khanh Linh[1], Kim Bao Giang[2]

[1] Department of Infectious Diseases, Hanoi Medical University, Hanoi, Vietnam, [2] Institute for Preventive Medicine and Public Health, Hanoi Medical University, Hanoi, Vietnam

Correspondence to: Vu Quoc Dat MD, PhD, Department of Infectious Diseases, Hanoi Medical University. E-mail: datvq@hmu.edu.vn/quocdat181@yahoo.com. Postal address: No 1 Ton That Tung street, Dong Da district, Hanoi, Vietnam.

Keywords: International Classification of Diseases, ICD-11, emergency, diagnosis, burden of disease, mortality, Vietnam

For peer review only

Abstract

Objective

The goal of this study was to describe the burden of disease and in-hospital mortality among patients admitted to the critical care units (CCUs) in Vietnam.

Design

Retrospective study.

Setting

The whole 1-year data of admissions to CCUs were collected from 34 hospitals from January to December 2018.

Participants

A total of 44,013 episodes of admission to CCUs were analysed.

Primary outcome

We used ICD-11 codes to assess the primary diagnosis associated with admissions and in-hospitals mortality. Years of life lost (YLL) measure was further used to estimate the burden of disease.

Results

The 0-5 years and ≥70 years age groups accounted for 14.8 % (6,508/44,013) and 26.1% (11,480/44,013) of all admissions, respectively. The most common diagnoses were diseases of the respiratory system (27.8% or 12,255/44,013), followed by unclassified symptoms, signs or clinical findings (13% or 5,712/44,013), and diseases of the circulatory system (12.2% or 5,380/44,013). Among 28,311 patients with available outcome data, 1,681 individuals (5.9%) died during the hospitalization. The in-hospital mortality rate increased with age, from 2.8% (86/3,105) in under 5 years old age group to 23.1% (297/1,288) in over 90-year age group. Diseases of the respiratory system was the leading causes of death in term of number of deaths (21.8% or 367/1,681 of all deaths). Diagnosis of sepsis was associated with the highest in-hospital mortality (36.8%). The overall YLL under the age of 75 were 1287 per 1000 patients.

For peer review only

Conclusions

CCUs in Vietnam faced wide differences in the burden of diseases. Sufficient infrastructure and adequate multidisciplinary training are essential to ensure the appropriate response to the current needs of population.

Strengths and limitations of this study

- We conducted a large multicentre study on the pattern of admission and frequency of diagnosis in the critical care units in Vietnam, a lower-middle income country.
- We present mortality, cause of admission and the years of life lost due to premature death in critical care units in provincial and district hospitals in Vietnam.
- The major limitation relates to the accuracy of the diagnosis and diagnostic coding in the study hospitals.

For peer review only

Introduction

Emergency care is an essential service of the hospitals which serves as a first point of contact to provide immediate treatment for individual with acute illness and life-threatening needs. Over 50% of mortality and over 40% of all morbidities worldwide can be contributed by emergency medical conditions [1]. In May 2019, the 63rd World Health Assembly recognized that existing data do not provide adequate support for effective planning and resource allocation for emergency care and urged member states to identify the local acute illness burden for improving quality of emergency care [2].

The International Statistical Classification of Diseases and Related Health Problems (often referred to as the International Classification of Diseases, ICD) has been used in more than 120 countries to monitor the incidence and prevalence of diseases [3]. Vietnam has implemented the ICD since 1998 as a tool for reporting morbidity, cause of death and billing for health insurance in hospitals. The burden of disease in Vietnam has changed rapidly over the past 2 decades with a marked shift toward the predominance of non-communicable diseases. Between 2000 and 2017, the share of communicable disease has reduced a half (24% to 14%), the share of non-communicable disease has increased almost 10% (63% to 74%) while the share of injuries still remained stable at around 12-13% (all measured in the disability-adjusted life year, DALY) [4]. However, the research specifically measuring the burden of diseases in emergency departments (EDs) or intensive care capable EDs (referred to as critical care units or CCUs) in Vietnam has been limited. Recently, some studies on the pattern of hospitalization, the causes of death or the hospital death certification have been performed [5-7], but none of these studies has provided a comprehensive description of the patterns of diagnosis or measured the premature death by the years of life lost (YLL) metric in patients with admission to CCUs in the country.

The lack of infrastructure and human resource brings significant challenges to the delivery of critical care in low- and middle-income countries (LMICs)[8]. The emergence of COVID-19 has put further pressure on the critical care system in these countries that already struggling with the high burden of admission to the CCUs in the pre-COVID-19 era. An understanding of the conditions that involve an admission to CCU will allows policymakers and planners to implement interventions that are effective in the national contexts to meet the care quality needs of the rapidly expanding aging populations and to prevent the

For peer review only - http://bmjopen.bmj.com/site/about/guidelines.xhtml

For peer review only

disruption of the critical care services in the pandemic. Our aim was to describe the frequency of diagnostic categories, in-hospital mortality, cause of death and the burden of premature mortality measuring by YLL in CCUs in Vietnam in 2018.

Methods

Study design and participants

This was a retrospective study of all episodes of CCU admissions to selected provincial and district hospitals in Vietnam from 1st January to 31st December 2018. We purposely selected 5 out of 63 provinces to represent 5 geographical areas in Vietnam. In each province, we invited all CCUs in hospitals in the catchment area.

Data were collected from the electronic hospital management system, including patient's demographic information, diagnosis, dates of admission, length of stay and outcome at discharge. Diagnosis of individual patients in each episode of admission were originally recorded in the ICD, 10th revision (ICD-10) codes. Because the ICD, 11th revision (ICD-11) came into effect in January 2022, for the monitoring of any change in the pattern of diseases responsible for admission in the future, all ICD-10 codes were translated to the ICD-11 codes (version 05/2021) using WHO mapping tables with the correspondence between ICD-10 and ICD-11 codes and link the ICD-11 codes with their corresponding associated descriptive labels. Two independent investigators (VQD and BTKL) manually reviewed any non-unique code or discrepancies in mapping between ICD-10 and ICD-11 which were eventually resolved by the consensus of two reviewers with the reference to the majority of code assignments by treating doctors.

The outcomes from the hospitalization were defined as death in patients who died in the hospital or was going home to die imminently after hospital discharge (palliative discharge). Palliative discharge is a common practice in Vietnam when a patient is in a moribund state and expresses a wish to die at home. Deterioration was defined as any worsening of clinical conditions by the treating doctor's judgment at the time of discharge. The data on the outcomes at CCU discharge and interventions during CCU stay was not available for analysis.

For peer review only - http://bmjopen.bmj.com/site/about/guidelines.xhtml

Statistical analysis

For the analysis of pattern of admission, we included all episodes of admission. Consequently, one patient might have multiple episodes of admission to CCUs during a year. For each episode, the primary diagnosis which was defined as the main reason for CCU admission was counted. The primary diagnosis for that episode may be the same or different from the previous episode of admission. Individual patients were identified within the datasets by matching on full name, gender, date of birth and insurance numbers when they were available). The in-hospital mortality was calculated by dividing the number of deaths by the total number of individual patients.

Years of life lost (YLLs) is a measure of premature mortality which is calculated from the number of deaths at each age multiplied by a standard life expectancy for the age at which death occurs [9]. Vietnam's standard life expectancy at birth in 2019 for both sexes were 73.7 years old [10] and to compare with other studies, we considered premature death were deaths under 75 years old. We also calculated the average YLL by dividing total of YLL by the number of premature death and the crude YLL rate by dividing total of YLL by the total number of patients aged under 75 years (expressed per 1000 persons).

We presented categorical variables as frequencies and percentages, while continuous variables as medians and interquartile ranges [IQR]. We used the chi-square test or Fisher's exact test to compare two binomial proportions. Statistical analyses were performed using SPSS version 27 (IBM, Armonk, NY, USA). Significant differences were considered if two-sided P values were < 0.05.

Patient and public involvement

Patients and the public were not involved in the design, recruitment or conduct of the study.

Results

Between 1 January 2018 and 31 December 2018, there were 44,013 admission episodes to CCUs from 34 hospitals in 5 provinces (namely Hanoi, Thai Nguyen, Ha Nam, Kon Tum and Can Tho), corresponding to 38534 unique patients. There were 3,647 (9.5%) patients with two or more admission in the study year (Figure 1). The median age was 53 years (interquartile range [IQR]) 25-70) and included 53.4% male. The majority of CCU admissions

For peer review only - http://bmjopen.bmj.com/site/about/guidelines.xhtml

in this study (31,569, 71.7%) were at the district hospitals. The median length of stay was 5 (IQR 2-8) days. A total of 1,764/36,518 admissions (4.8%) had a length of stay of more than 14 days. The hospitalization outcome was available for 28,311/38,534 (73.5%) patients. Overall survival to hospital discharge was 94.1% (26,630/28,311).

Admission diagnosis in critical care units

Of 44,013 episodes of admission to CCUs, the most common diagnosis were respiratory diseases (12,255 episodes, 27.8%) in which, 38.7% was certain lower respiratory tract diseases (blockL1-CA2) and 34.4% was lung infections (blockL1-CA4), followed by unclassified symptoms, signs or clinical findings (5,712 episodes or 13.0%) (51.8% was of digestive system or abdomen, blockL1-MD8), circulatory system diseases (5,380 episodes, 12.2%) (51,2% was hypertensive diseases, blockL1-BA0), diseases of the nervous system (5,019 episodes, 11.4%) (85.4% was cerebrovascular diseases, blockL1-8B0) and injury, poisoning or consequences of external causes (4,186 episodes or 9.5%) (39.7% was harmful effect off substances, blockL1-NE6). The percentage of diagnosis in episodes of CCU admission varied by age groups (Figure 2). The number of admissions peaked in children from 5 years old and younger and was lowest in the 5-10 years age group (Figure 3). The respiratory disease was the leading cause of admission in the under-10-years age group (64% of admissions) and age groups of 61 years and older (29% of admissions) (Figure 2)

The number of admissions in all study hospitals fluctuated across months but mostly driven by the admission by respiratory diseases (Supplementary Figure 1). The proportions of admission to CCUs were significantly higher in provincial hospitals than in district hospital in regard to diseases of the nervous system (18.7% vs 8.5%, p<0.001), certain infectious or parasitic diseases (8.9% vs 3.4%, p<0.001), injury/poisoning (10.3% vs 9.2%, p=0.00), endocrine, nutritional or metabolic diseases (3.6% vs 2.2%, p<0.001) and neoplasms (2% vs 0.7%, p<0.001) (Figure 4**Error! Reference source not found.**).

Outcome of hospitalization

The median length of stay for all admissions was 5 days (IQR 2-8). Among 28,311 patients with available outcome data and, 1,681/28,311 (5.9%) died. The median age of death was 68 years (IQR 47-86) and 63.3% of death were in men. Men had significantly higher in-hospital mortality rate than women (3.8% vs. 2.6%, p<0.001). The median duration of hospital stay for the survivors and death cases was 4 (IQR:2-8) and 3 (IQR: 2–7), respectively.

For peer review only - http://bmjopen.bmj.com/site/about/guidelines.xhtml

More than three quarters of deaths (1,290/1,681 or 76.7%) occurred in provincial hospitals. The all-cause in-hospital mortality rates by age groups and mortality rate for selected groups of diagnosis are presented in Figure 3 and Supplementary Table 1. The most common causes of death by ICD-11 block codes and chapter codes are showed in Table 1 and Supplementary Table 2, respectively.

For peer review only

For peer review only - http://bmjopen.bmj.com/site/about/guidelines.xhtml

Table 1. The top 20 causes of death in terms of years of life lost (YLL) and numbers of deaths by ICD-11 block level 1 codes

Diagnosis	Number and proportion of death cases	In-hospital mortality (n,%)	Total YLL75	Proportion of YLLs75	YLL75 per 1000 patients	Length of stay to death (median, IQR)
Cerebrovascular diseases	303 (18.0%)	**8.7%**	3211000	**12.3%**	1380	3 (2-6)
Miscellaneous	259 (15.4%)	**2.3%**	5686000	**38.2%**	614	2 (1-6)
Lung infections	201 (12.0%)	**7.2%**	2875000	**9.5%**	1440	7 (3-15)
Sepsis	149 (8.9%)	**36.8%**	1991000	**2.6%**	8127	4 (2-11)
Harmful effects of substances	87 (5.2%)	**6.1%**	3534000	**5.0%**	2544	1 (1-2)
Respiratory failure	79 (4.7%)	**18.1%**	365000	**1.6%**	1714	9 (5-15)
General symptoms, signs or clinical findings	67 (4.0%)	**13.5%**	1507000	**2.2%**	3712	2 (1-4.5)
Injuries to the head	66 (3.9%)	**15.6%**	1140000	**1.6%**	3167	2 (2-4)
Certain lower respiratory tract diseases	63 (3.7%)	**2.1%**	443000	**10.2%**	217	4 (2-10)
Symptoms, signs or clinical findings of the digestive system or abdomen	61 (3.6%)	**5.5%**	994000	**4.0%**	1062	3 (2-6)
Malignant neoplasms, except primary neoplasms of lymphoid, haematopoietic, central nervous system or related	50 (3.0%)	**18.0%**	587000	**1.0%**	3073	6 (3-9)
Symptoms, signs or clinical findings of the circulatory system	50 (3.0%)	**31.8%**	1060000	**0.7%**	8217	2 (1-6)
Heart failure	39 (2.3%)	**8.4%**	280000	**1.6%**	1466	3 (1-5)
Ischaemic heart diseases	37 (2.2%)	**5.2%**	78000	**2.5%**	158	3 (1-5)
Diseases of liver	36 (2.1%)	**11.2%**	549000	**1.1%**	1861	2.5 (1.5-7.5)
Injuries involving multiple body regions	29 (1.7%)	**28.2%**	439000	**0.4%**	6362	2 (1-6)
Symptoms, signs or clinical findings of the respiratory system	28 (1.7%)	**8.4%**	706000	**1.1%**	3017	2 (1-4)
Mental or behavioural symptoms, signs or clinical findings	22 (1.3%)	**13.3%**	363000	**0.8%**	2771	2 (1-2)
Allergic or hypersensitivity conditions	20 (1.2%)	**3.3%**	890000	**2.2%**	1537	2 (1-2)
Infection, unspecified	19 (1.1%)	**31.7%**	741000	**0.2%**	14529	2 (2-4)
Epilepsy or seizures	16 (1.0%)	**5.0%**	696000	**1.1%**	2275	1.5 (1-2)
Total	1681 (100.0%)	**5.9%**	28135000	**100.0%**	1287	3 (2-7)

For peer review only - http://bmjopen.bmj.com/site/about/guidelines.xhtml

The in-hospital mortality in patients over 75 years was 662/6,140 (10.8%). In total, 28,135,000 years were lost among patient admitted to the study CCUs. This yielded an overall YLL under the age of 75 years of 1,287 per 1,000 people. Table 1 presents the top 10 causes of death by ICD-11 block codes. Cerebrovascular diseases rank the first in the number of deaths (303/1,681 or 18%) and the eleven when measuring in in-hospital mortality (8.7%) while sepsis ranked as the first specified cause of death in terms of in-hospital mortality (36.8%) and ranked fourth in term of death counts.

Discussion

This study presents for the first time, data to quantify the burden of diseases in CCUs, for the most common reasons of admission and the in-hospital mortality of patients admitted to CCUs in Vietnam. Few studies on admission to the CCUs in lower-middle income countries have been reported earlier.

Burden of diseases in Vietnam has changed significantly in the past 20 years [4]. The communicable diseases burden (measured in disability-adjusted life years) decreased rapidly from 37% in 1990 to 14% in 2017. However, in the critical care settings, our study shows that the infectious diseases remain a major emergency medical condition and are the prime cause of morbidity and mortality in terms of number of admission and in terms of YLL.

Our findings on the difference distribution of diseases in CCUs in provincial and district hospitals reflected the variation of healthcare services and capacity of clinical management. In 2019, we conducted a survey on the capacity and use of diagnostics and treatment for patients with severe acute respiratory infections in 48 hospitals in Vietnam (including 32 hospitals in this study) at the same time as this study. Significant differences were observed in the district hospitals when compared to provincial hospitals in regards to availability of the microbiological diagnostics (9.4% vs 81.2%, $p<0.001$), inflammatory markers of C-reactive protein (31.2% vs 54.2%, $p<0.001$) and procalcitonin (3.1% vs 25%, $p<0.001$) and mechanical ventilation (40.6% vs 60.4%, $p<0.001$)[11]. Provincial hospitals in Vietnam are the secondary hospitals which do not only treat referred patients from lower level of care (district hospitals or commune centres) but also admit patients from the community. Additionally, patient can directly self-refer to higher level of hospitals which resulting in

For peer review only - http://bmjopen.bmj.com/site/about/guidelines.xhtml

under-utilization of district hospitals and overcrowding in provincial hospitals, especially for given specialities which requires higher levels of skills and capacity.

Our findings are partly aligned with previous reports in all-cause mortality in both sexes and all ages showing that cardiovascular (ischaemic heart disease, stroke), respiratory diseases and injuries were the significant challenge. WHO's report in 2019 showed that ischaemic heart disease, stroke, neonatal conditions, chronic obstructive pulmonary disease (COPD) and lower respiratory infections were the top 5 causes of death in term of death counts in LMICs [12]. At the global level in 2019, the top three diseases with highest burden (measuring in DALY) were neonatal disorder (32.9% of global DALYs), lower respiratory infections (11% of global DALYs) and diarrhoeal diseases (9.3% of global DALYs) in children younger than 10 years and were ischemic heart diseases (16.2% of global DALY), stroke (13% of global DALY) and COPD (8.5% of global DALY) in patients from 75 years and older [13]. In Vietnam, stroke (164.8 deaths per 100,000), ischaemic heart disease (95.3 deaths per 100,000), COPD (37.2 deaths per 100,000) and diabetes mellitus (35.1 deaths per 100,000) and road injuries (30.5 deaths per100,000) were in the top-five rankings for total number of death in 2019 [14]. Sepsis and harmful effects of substances were not listed in the top 5 cause of death in the general population, but we found that these were important conditions in emergency care settings.

The overall mortality in our study were higher than previous report. In a systematic review of data from 65 emergency departments in 59 LMICs countries between 2013 and 2014, the median mortality was 1.8% (IQR 0.5-5.1%), highest in sub-Saharan Africa (median 3.4%, IQR: 0.5–6.3%) and lowest in South Asia, East Asia & Pacific (0.3, IQR 0.2–0.8%) [15].

In our study, patients aged 75 years or older presented 18.9% of all admission at CCUs despite the fact that they comprised 3.2% of general population [16]. The percentage of population older than 75 years in Vietnam is expected to increase to 4.2% by 2030 and 6.6% by 2040 [16]. This will increase the demand for critical care resources and requires further evaluation to guide healthcare decision-making and changes in organizational models in CCU and delivery of critical care services during a pandemic.

The design of multicentre study, large population and data collection for the whole year of 2018 are the strengths of our study. The major limitations related to the diagnosis

For peer review only - http://bmjopen.bmj.com/site/about/guidelines.xhtml

description and diagnostic coding by doctors in study hospitals. Data quality of diagnosis may vary between hospitals due to the accurate clinical diagnosis of disease depends on doctor's knowledge and skills, available laboratory investigations and diagnostic imaging procedures. However, there are differences in the capacity of clinical management and use of diagnostics between provincial and district hospitals in the country [11]. In Vietnam, treating doctors are responsible for assigning the ICD diagnostic code to each patient discharged from the hospital. The complex nature of ICD coding, patient's condition in critical care settings and the lack of coding experience pose further challenges for the accuracy and reliability of the diagnosis given across hospitals. Another limitation was the missing information on outcome for 26.5% (10,223/38543) of patients because these were not actually entered into the electronic hospital management system. This missing data can cause bias in the estimation of in-hospital mortality.

Currently, there is limited cost-effectiveness analysis performed for critical care services in Vietnam. The results of this study in regard to the variety of diagnoses and outcomes of admission will direct the future studies on the establishment of CCU registry, the effectiveness of CCU treatments and estimates of the total cost to save a life-year. This information will be helpful to develop policies and strategies to make better-informed decisions in resource-constrained settings in the face of an aging population in Vietnam.

Conclusion

Our study indicates a variation of needs of emergency care by age groups and levels of care among patients admitted to CCUs in provincial and district hospitals in Vietnam. These findings support the need to provide multidisciplinary team training for clinicians in CCUs and the future studies should further assess the emergency and critical care capacity needs, ability of the healthcare setting to provide the emergency services and the quality of care (including minimal set of indicators) and gaps to mobilize the resources required for live saving during a pandemic.

Data availability statement

Data are available upon reasonable request.

For peer review only - http://bmjopen.bmj.com/site/about/guidelines.xhtml

Patient consent for publication

Not applicable.

Ethics approval

This study was approved by the institutional review board (IRB) in the Hanoi Medical University (59/GCN-DDNCYSH-DHYHN).

Funding

This work was supported by Viet Nam - WHO Country Office. The funder of the study had no role in study design, data collection, data analysis, data interpretation, or writing of the report.

Competing interests

We declare no competing interest.

Contributors

VQD conceived and designed the study. VQD analysed the data and wrote the first draft of the report. VQD, BTKL and KBG involved to the acquisition and interpretation of data. All authors contributed to and approved the final report.

Acknowledgments

We are very grateful to the patients and staff at the participating hospitals in Ha Noi, Thai Nguyen, Ha Nam, Kon Tum and Can Tho. We would like to thank Drs. Satoko Otsu and Vu Quang Hieu (World Health Organization, Vietnam Country Office) for their tireless support and contribution to strengthening the capacity for clinicians in Vietnam and thank Dr. Nguyen The Hung (National Hospital for Tropical Diseases) for his initial support in the implementation of this study.

REFERENCES

1. Razzak J, Usmani MF, Bhutta ZA. Global, regional and national burden of emergency medical diseases using specific emergency disease indicators: analysis of the 2015 Global Burden of Disease Study. *BMJ Glob Health* 2019;4(2):e000733. doi: 10.1136/bmjgh-2018-000733
2. World Health Assembly. Emergency care systems for universal health coverage: ensuring timely care for the acutely ill and injured. Geneva: World Health Organization, 2019.

For peer review only - http://bmjopen.bmj.com/site/about/guidelines.xhtml

3. Executive Board. International Classification of Diseases: International Statistical Classification of Diseases and Related Health Problems: update on the eleventh revision: report by the Director-General. Geneva: World Health Organization, 2018.
4. Elena Glinskaya, Annette De Kleine Feige, Vu Thi Lan Hoang, et al. Vietnam - Adapting to an Aging Society Washington, D.C: World Bank Group, 2021.
5. Nguyen NTT, Dien TM, Schindler C, et al. Childhood hospitalisation and related deaths in Hanoi, Vietnam: a tertiary hospital database analysis from 2007 to 2014. *BMJ open* 2017;7(7):e015260. doi: 10.1136/bmjopen-2016-015260
6. Hong TT, Phuong Hoa N, Walker SM, et al. Completeness and reliability of mortality data in Viet Nam: Implications for the national routine health management information system. *PloS one* 2018;13(1):e0190755. doi: 10.1371/journal.pone.0190755
7. Walton M, Harrison R, Chevalier A, et al. Improving hospital death certification in Vietnam: results of a pilot study of injury-related fatalities. *Inj Prev* 2018;24(5):324-31. doi: 10.1136/injuryprev-2017-042341
8. Murthy S, Leligdowicz A, Adhikari NK. Intensive care unit capacity in low-income countries: a systematic review. *PloS one* 2015;10(1):e0116949. doi: 10.1371/journal.pone.0116949
9. World Health Organization. WHO methods and data sources for global burden of disease estimates 2000-2019. In: Analytics DoDa, Division of Data AaDfI, eds. Geneva: World Health Organization,, 2020.
10. World Health Organization. Life expectancy and Healthy life expectancy. Data by country 2020 [updated 4th December 2021; cited 2022 6th January]. Available from: https://apps.who.int/gho/data/node.main.688 accessed 6th January 2022.
11. Dat VQ, Hung NT, Giang BK, et al. Capacity and use of diagnostics and treatment for patients with severe acute respiratory infections in the pre-COVID-19 era in district and provincial hospitals in Viet Nam. *Western Pac Surveill Response J* 2021;12(4):1-9. doi: doi.org/10.5365/wpsar.2021.12.4.835
12. World Health Organization. The top 10 causes of death 2019 [updated 9th December 2020; cited 2021 11th December]. Available from: https://www.who.int/news-room/fact-sheets/detail/the-top-10-causes-of-death accessed 11th December 2021.
13. GBD 2019 Diseases and Injuries Collaborators. Global burden of 369 diseases and injuries in 204 countries and territories, 1990-2019: a systematic analysis for the Global Burden of Disease Study 2019. *Lancet* 2020;396(10258):1204-22. doi: 10.1016/S0140-6736(20)30925-9
14. World Health Organization. Global Health Estimates 2020: Deaths by Cause, Age, Sex, by Country and by Region, 2000-2019 2020 [cited 2021 13th December]. Available from: https://www.who.int/data/gho/data/themes/mortality-and-global-health-estimates/ghe-leading-causes-of-death accessed 13th December 2021.
15. Obermeyer Z, Abujaber S, Makar M, et al. Emergency care in 59 low- and middle-income countries: a systematic review. *Bull World Health Organ* 2015;93(8):577-86G. doi: 10.2471/BLT.14.148338
16. General Statistic Office, United Nations Population Fund. Vietnam population projection for the period 2019-2069. In: Office GS, ed. Hanoi, 2020.

Figure Titles

Figure 1. Flowchart of CCU admission and outcome

Figure 2. Diagnosis on admission to CCU by age groups

Figure 3. Number of admission and In-hospital mortality by age groups

Figure 4. Cause of admission to CCUs by level of care

For peer review only - http://bmjopen.bmj.com/site/about/guidelines.xhtml

Supplementary materials

Supplementary Figure 1. Pattern of admissions

Supplementary Table 1. Mortality and years of life lost (YLL) grouped by ICD-11 chapter codes

Supplementary Table 2. Number of deaths by ICD-11 4-character codes

For peer review only

For peer review only - http://bmjopen.bmj.com/site/about/guidelines.xhtml

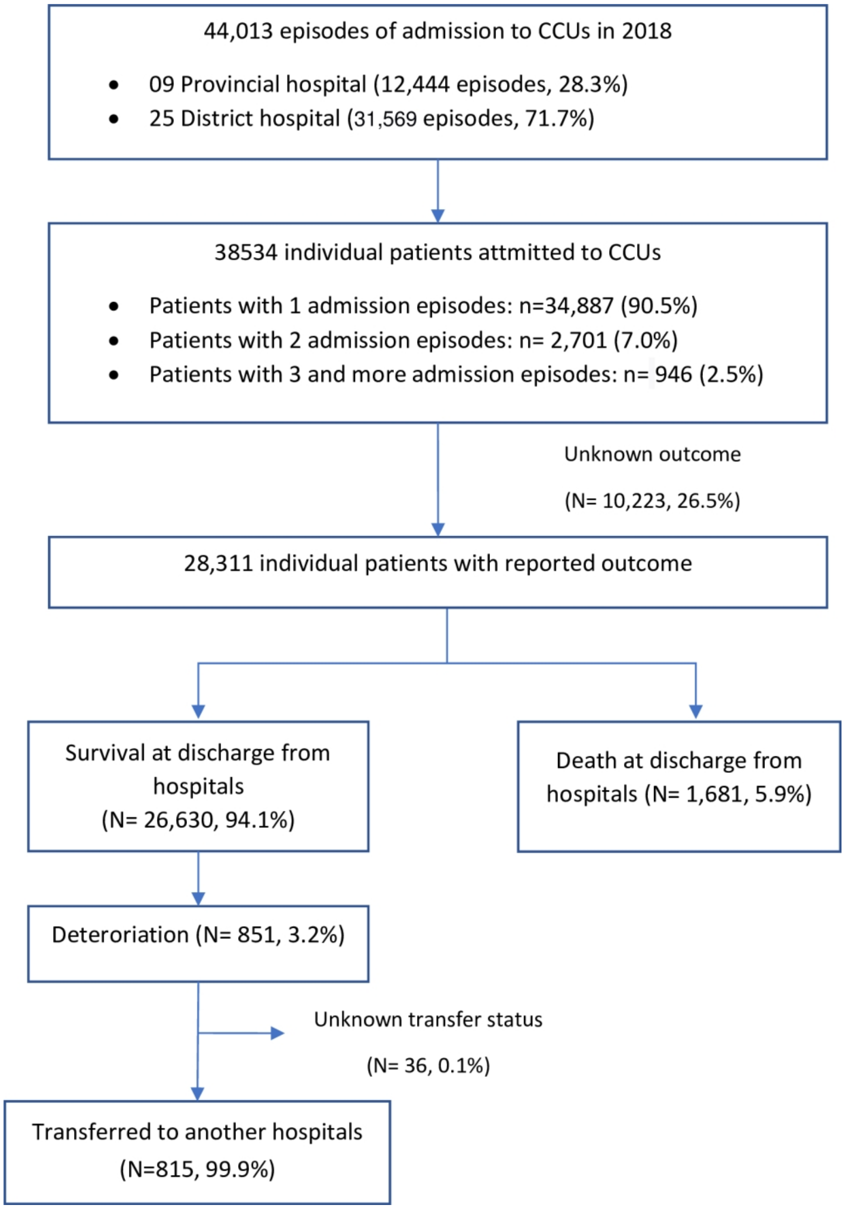

Figure 1. Flowchart of CCU admission and outcome.

129x185mm (300 x 300 DPI)

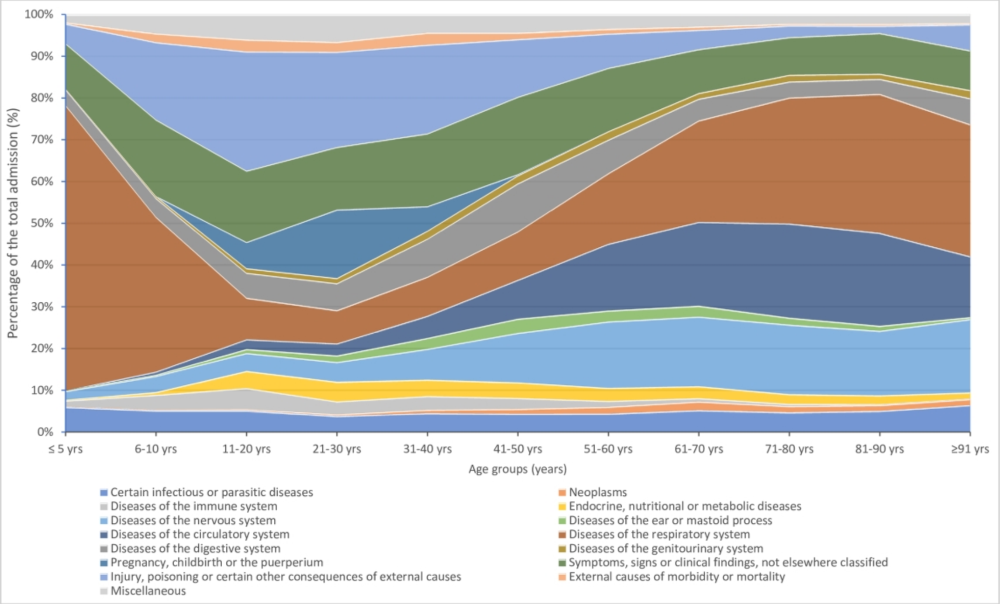

Figure 2. Diangosis on admission to CCU by age groups.

190x115mm (300 x 300 DPI)

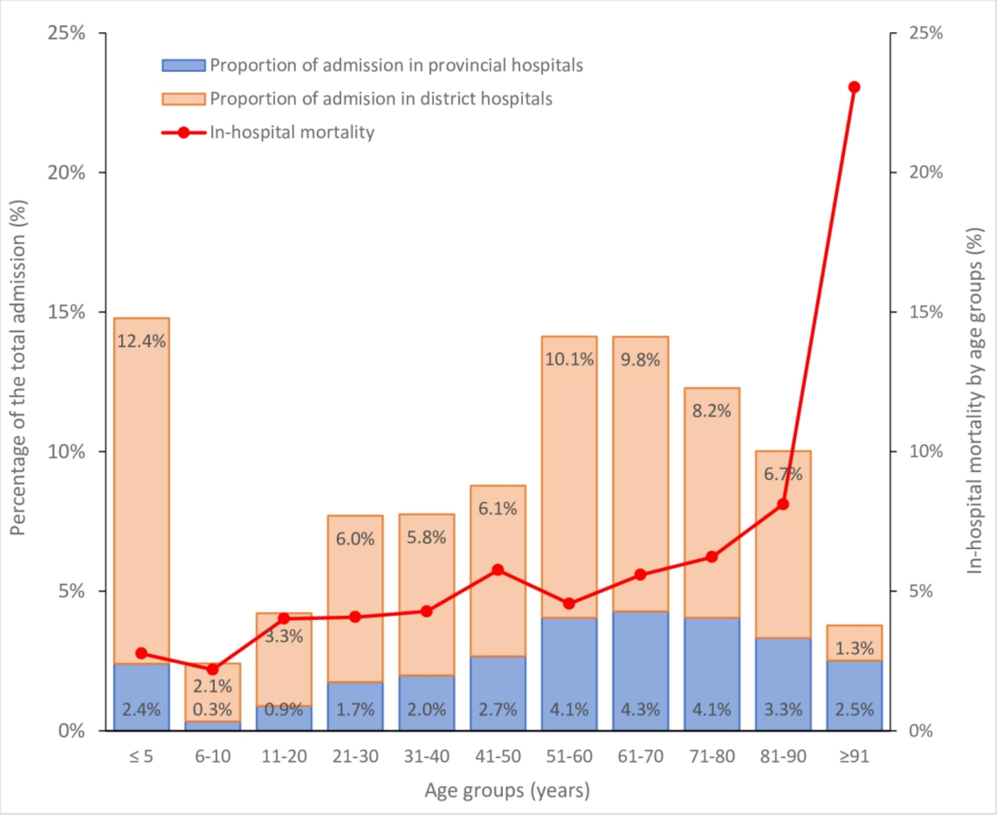

Figure 3. Number of admission and In-hospital mortality by age groups

159x129mm (300 x 300 DPI)

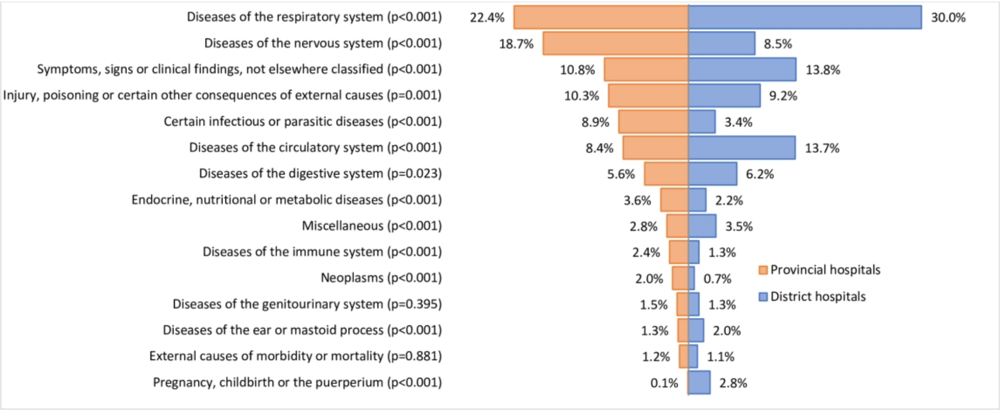

Figure 4. Cause of admission to CCUs by level of care.

190x77mm (300 x 300 DPI)

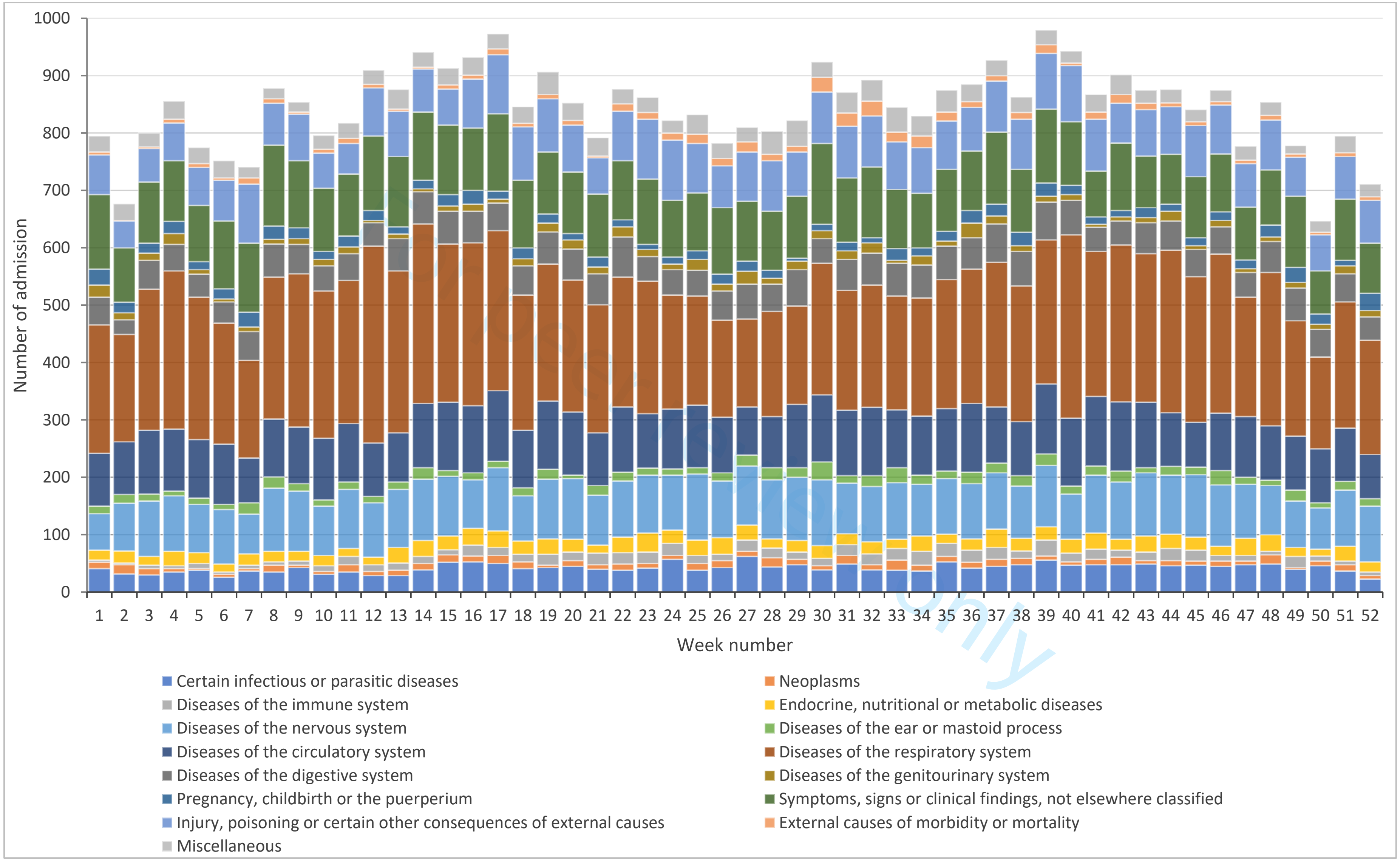

Supplementary Figure 1. Pattern of admissions over months.

Supplementary Table 1. Mortality and years of life lost (YLL) grouped by ICD-11 chapter codes

Diagnosis	Number and proportion of death cases	In-hospital mortality (n,%)	Total YLL75	Proportion of YLLs75	YLL75 per 1000 patients	Length of stay to death (median, IQR)
Diseases of the respiratory system	367 (21.8%)	4.8%	4506000	25.9%	813	6 (3-13)
Diseases of the nervous system	324 (19.3%)	8.1%	3994000	14.0%	1432	2 (2-6)
Symptoms, signs or clinical findings, not elsewhere classified	241 (14.3%)	9.4%	5141000	9.8%	2476	2 (1-4)
Injury, poisoning or certain other consequences of external causes	210 (12.5%)	8.4%	5846000	8.8%	2557	2 (1-2)
Certain infectious or parasitic diseases	188 (11.2%)	14.0%	3218000	5.8%	3056	4 (2-10)
Diseases of the circulatory system	112 (6.7%)	2.8%	817000	13.4%	314	3 (1-5)
Diseases of the digestive system	83 (4.9%)	3.9%	1221000	7.1%	683	3 (2-8)
Neoplasms	59 (3.5%)	17.3%	809000	1.3%	3385	5 (2-9)
Miscellaneous	31 (1.8%)	3.5%	963000	3.1%	1259	2 (1-5)
Endocrine, nutritional or metabolic diseases	22 (1.3%)	2.4%	217000	3.3%	276	2 (1-3)
Diseases of the immune system	20 (1.2%)	3.3%	890000	2.2%	1532	2 (1-2)
Diseases of the genitourinary system	13 (0.8%)	3.0%	168000	1.5%	487	5 (2-11)
External causes of morbidity or mortality	8 (0.5%)	1.8%	223000	1.6%	556	3.5 (1.5-4.5)
Diseases of the ear or mastoid process	2 (0.1%)	0.4%	87000	1.8%	192	1 (1-1)
Pregnancy, childbirth or the puerperium	1 (0.1%)	0.7%	35000	0.5%	241	1 (1-1)
Total	1681 (100.0%)	5.9%	28135000	100.0%	1287	3 (2-7)

For peer review only

Supplementary Table 2. The number of deaths by ICD 4-character codes

	Causes of death	N (%)
CHAPTER 01 Certain infectious or parasitic diseases	Viral intestinal infections, unspecified	1 (0.06%)
	Gastroenteritis or colitis without specification of infectious agent	1 (0.06%)
	Tuberculosis of the respiratory system	2 (0.12%)
	Bacterial cellulitis, erysipelas and lymphangitis	1 (0.06%)
	Other specified pyogenic bacterial infection of skin and subcutaneous tissue	1 (0.06%)
	Tetanus	1 (0.06%)
	Bacterial infection of unspecified site	1 (0.06%)
	Viral encephalitis not elsewhere classified	2 (0.12%)
	Viral meningitis not elsewhere classified	1 (0.06%)
	Infectious abscess of the central nervous system	1 (0.06%)
	Dengue fever, unspecified	1 (0.06%)
	Acute viral hepatitis	4 (0.24%)
	Viral hepatitis, unspecified	2 (0.12%)
	Phaeohyphomycosis	1 (0.06%)
	Sepsis without septic shock	23 (1.37%)
	Sepsis with septic shock	126 (7.50%)
	Infection, unspecified	19 (1.13%)
CHAPTER 02 Neoplasms	Primary neoplasms of brain	6 (0.36%)
	Malignant haematopoietic neoplasms without further specification	1 (0.06%)
	Malignant neoplasms of other or ill-defined sites in the lip, oral cavity or pharynx	2 (0.12%)
	Malignant neoplasms of oesophagus	5 (0.30%)
	Malignant neoplasms of stomach	2 (0.12%)
	Malignant neoplasms of colon	5 (0.30%)
	Malignant neoplasm of pancreas	1 (0.06%)
	Malignant neoplasms of liver or intrahepatic bile ducts	11 (0.65%)
	Malignant neoplasms of gallbladder	1 (0.06%)
	Malignant neoplasms of bronchus or lung	10 (0.59%)
	Malignant neoplasms of thymus	1 (0.06%)
	Malignant neoplasms of heart, mediastinum or non-mesothelioma of pleura	2 (0.12%)
	Malignant neoplasms of breast, unspecified	3 (0.18%)
	Malignant neoplasms of prostate	1 (0.06%)
	Malignant neoplasms of bladder	1 (0.06%)
	Malignant neoplasms of thyroid gland	1 (0.06%)
	Malignant neoplasm metastasis in lung	4 (0.24%)
	Carcinoma in situ of bladder	1 (0.06%)
	Benign osteogenic tumours	1 (0.06%)
CHAPTER 03 Diseases of the blood or blood-forming organs	Anaemia due to chronic disease	1 (0.06%)
	Anaemias or other erythrocyte disorders, unspecified	8 (0.48%)
	Anaphylaxis	9 (0.54%)

For peer review only

	Causes of death	N (%)
**CHAPTER 04 Diseases of the immune system**	Allergic or hypersensitivity conditions of unspecified type	11 (0.65%)
**CHAPTER 05 Endocrine, nutritional or metabolic diseases**	Type 1 diabetes mellitus	3 (0.18%)
	Type 2 diabetes mellitus	3 (0.18%)
	Hypoglycaemia without associated diabetes	5 (0.30%)
	Increased secretion of glucagon	1 (0.06%)
	Protein deficiency	1 (0.06%)
	Hyperosmolality or hypernatraemia	1 (0.06%)
	Acidosis	1 (0.06%)
	Hyperkalaemia	1 (0.06%)
	Hypokalaemia	1 (0.06%)
	Disorders of fluid, electrolyte or acid-base balance, unspecified	5 (0.30%)
**CHAPTER 06 Mental, behavioural or neurodevelopmental disorders**	Developmental learning disorder	1 (0.06%)
	Dissociative disorders, unspecified	4 (0.24%)
	Disorders due to use of dissociative drugs including ketamine and phencyclidine [PCP]	1 (0.06%)
	Delirium	1 (0.06%)
	Mental, behavioural or neurodevelopmental disorders, unspecified	2 (0.12%)
**CHAPTER 08 Diseases of the nervous system**	Seizure due to acute causes	2 (0.12%)
	Types of seizures	11 (0.65%)
	Epilepsy or seizures, unspecified	3 (0.18%)
	Intracerebral haemorrhage	133 (7.91%)
	Subarachnoid haemorrhage	3 (0.18%)
	Intracranial haemorrhage, unspecified	32 (1.90%)
	Cerebral ischaemic stroke	72 (4.28%)
	Stroke not known if ischaemic or haemorrhagic	61 (3.63%)
	Late effects of cerebrovascular disease	2 (0.12%)
	Myelitis	1 (0.06%)
	Motor neuron disease	1 (0.06%)
	Hereditary sensory or autonomic neuropathy	1 (0.06%)
	Increased intracranial pressure	1 (0.06%)
	Disorders of the meninges excluding infection	1 (0.06%)
**CHAPTER 10 Diseases of the ear or mastoid process**	Disorders of vestibular function	2 (0.12%)
**CHAPTER 11 Diseases of the circulatory system**	Essential hypertension	8 (0.48%)
	Hypertensive heart disease	1 (0.06%)
	Hypotension, unspecified	5 (0.30%)
	Angina pectoris	1 (0.06%)
	Acute myocardial infarction	36 (2.14%)
	Pulmonary thromboembolism	1 (0.06%)
	Pulmonary hypertension	1 (0.06%)
	Pulmonary heart disease or diseases of pulmonary circulation, unspecified	5 (0.30%)
	Pericardial effusion	1 (0.06%)
	Mitral valve stenosis with insufficiency	1 (0.06%)

For peer review only

	Causes of death	N (%)
	Myocarditis	1 (0.06%)
	Conduction disorders	1 (0.06%)
	Ventricular tachyarrhythmia	2 (0.12%)
	Cardiac arrhythmia, unspecified	5 (0.30%)
	Congestive heart failure	3 (0.18%)
	Heart failure, unspecified	36 (2.14%)
	Acute arterial occlusion	1 (0.06%)
	Aortic aneurysm or dissection	1 (0.06%)
	Diseases of arteries or arterioles, unspecified	1 (0.06%)
	Deep vein thrombosis	1 (0.06%)
**CHAPTER 12 Diseases of the respiratory system**	Acute tonsillitis	1 (0.06%)
	Acute laryngitis or tracheitis	1 (0.06%)
	Acute upper respiratory infections of multiple and unspecified sites	1 (0.06%)
	Bronchitis	2 (0.12%)
	Chronic obstructive pulmonary disease	52 (3.09%)
	Asthma	8 (0.48%)
	Bronchiectasis	1 (0.06%)
	Pneumonia	198 (11.78%)
	Abscess of lung or mediastinum	2 (0.12%)
	Lung infections, unspecified	1 (0.06%)
	Pneumonitis due to solids and liquids	1 (0.06%)
	Respiratory conditions due to inhalation of chemicals, gases, fumes or vapours	3 (0.18%)
	Acute respiratory distress syndrome	2 (0.12%)
	Pulmonary oedema	11 (0.65%)
	Pneumothorax	2 (0.12%)
	Haemothorax	1 (0.06%)
	Pleural effusion	1 (0.06%)
	Respiratory failure	79 (4.70%)
**CHAPTER 13 Diseases of the digestive system**	Gastro-oesophageal reflux disease	1 (0.06%)
	Vascular disorders of the oesophagus	3 (0.18%)
	Gastritis	4 (0.24%)
	Gastric ulcer	5 (0.30%)
	Obstruction of small intestine	7 (0.42%)
	Appendicitis	3 (0.18%)
	Obstruction of large intestine	1 (0.06%)
	Polyp of large intestine	1 (0.06%)
	Infectious liver disease	1 (0.06%)
	Acute or subacute hepatic failure	2 (0.12%)
	Hepatic fibrosis or cirrhosis	15 (0.89%)
	Autoimmune liver disease	1 (0.06%)
	Certain specified diseases of liver	17 (1.01%)
	Cholelithiasis	2 (0.12%)
	Cholangitis	2 (0.12%)
	Acute pancreatitis	8 (0.48%)
	Peritonitis	6 (0.36%)
	Inguinal hernia	1 (0.06%)
	Indeterminate colitis	1 (0.06%)

For peer review only

	Causes of death	N (%)
	Diseases of the digestive system, unspecified	2 (0.12%)
**CHAPTER 14 Diseases of the skin**	Lichen planus	1 (0.06%)
	Skin disease of unspecified nature	1 (0.06%)
**CHAPTER 16 Diseases of the genitourinary system**	Pyonephrosis	1 (0.06%)
	Acute kidney failure	1 (0.06%)
	Chronic kidney disease	7 (0.42%)
	Kidney failure, unspecified	2 (0.12%)
	Urinary tract infection, site not specified	2 (0.12%)
**CHAPTER 18 Pregnancy, childbirth or the puerperium**	Complications following abortion, ectopic or molar pregnancy	1 (0.06%)
**CHAPTER 19 Certain conditions originating in the perinatal period**	Sepsis of foetus or newborn	1 (0.06%)
	Respiratory failure of newborn	1 (0.06%)
	Transitory disorders of carbohydrate metabolism specific to foetus or newborn	1 (0.06%)
	Certain specified transitory neonatal electrolyte or metabolic disturbances	1 (0.06%)
**CHAPTER 20 Developmental anomalies**	Congenital hydrocephalus	1 (0.06%)
	Congenital anomaly of a ventricle or the ventricular septum	1 (0.06%)
	Structural developmental anomalies of gallbladder, bile ducts or liver	1 (0.06%)
**CHAPTER 21 Symptoms, signs or clinical findings, not elsewhere classified**	Symptoms, signs or clinical findings involving consciousness	22 (1.31%)
	Abnormalities of heart beat	2 (0.12%)
	Cardiac arrest	48 (2.86%)
	Abnormalities of breathing	2 (0.12%)
	Haemorrhage from throat	1 (0.06%)
	Haemoptysis	2 (0.12%)
	Respiratory arrest	23 (1.37%)
	Abdominal or pelvic pain	4 (0.24%)
	Nausea or vomiting	1 (0.06%)
	Clinical manifestations of the digestive system	56 (3.33%)
	Cachexia	1 (0.06%)
	Fever of other or unknown origin	4 (0.24%)
	Haemorrhage, not elsewhere classified	1 (0.06%)
	Shock	60 (3.57%)
	Syncope and collapse	1 (0.06%)
	Other sudden death, cause unknown	1 (0.06%)
	Other ill-defined and unspecified causes of mortality	9 (0.54%)
	Multi organ failure	3 (0.18%)
**CHAPTER 22 Injury, poisoning or certain other consequences of external causes**	Injury of cranial nerves	1 (0.06%)
	Intracranial injury	65 (3.87%)
	Superficial injury of thorax	2 (0.12%)
	Injury of blood vessels at abdomen, lower back or pelvis level	1 (0.06%)
	Injury of intra-abdominal organs	5 (0.30%)
	Traumatic amputation of forearm	1 (0.06%)

For peer review only

	Causes of death	N (%)
	Fracture of femur	1 (0.06%)
	Superficial injury of ankle or foot	1 (0.06%)
	Crushing injuries involving multiple body regions	1 (0.06%)
	Unspecified multiple injuries	28 (1.67%)
	Burn of wrist or hand	1 (0.06%)
	Burns of external body surface, other specified site	1 (0.06%)
	Burns of multiple body regions	1 (0.06%)
	Harmful effects of drugs, medicaments or biological substances, not elsewhere classified	57 (3.39%)
	Harmful effects of or exposure to noxious substances, chiefly nonmedicinal as to source, not elsewhere classified	30 (1.78%)
	Effects of other specified external causes	3 (0.18%)
	Certain early complications of trauma, not elsewhere classified	11 (0.65%)
**CHAPTER 23 External causes of morbidity or mortality**	Unintentional exposure to or harmful effects of pesticides	1 (0.06%)
	Intentional self-harm by threat to breathing by suffocation from object covering mouth or nose	1 (0.06%)
	Intentional self-harm by and exposure to other and unspecified drug, medicament and biological substance	3 (0.18%)
	Intentional self-harm by exposure to or harmful effects of pesticides	1 (0.06%)
	Intentional self-harm by exposure to or harmful effects of other or unspecified substances chiefly nonmedicinal as to so	1 (0.06%)
	Exposure to or harmful effects of undetermined intent of other or unspecified drugs, medicaments or biological substance	1 (0.06%)
**CHAPTER 24 Factors influencing health status or contact with health services**	Surgical or postsurgical states, unspecified	3 (0.18%)
	Personal history of malignant neoplasm	1 (0.06%)

For peer review only

STROBE Statement—Checklist of items that should be included in reports of ***cross-sectional studies***

	Item No	Recommendation	Page No
**Title and abstract**	1	(*a*) Indicate the study's design with a commonly used term in the title or the abstract	1
		(*b*) Provide in the abstract an informative and balanced summary of what was done and what was found	2
**Introduction**			
Background/rationale	2	Explain the scientific background and rationale for the investigation being reported	4
Objectives	3	State specific objectives, including any prespecified hypotheses	5
**Methods**			
Study design	4	Present key elements of study design early in the paper	5
Setting	5	Describe the setting, locations, and relevant dates, including periods of recruitment, exposure, follow-up, and data collection	5
Participants	6	(*a*) Give the eligibility criteria, and the sources and methods of selection of participants	5
Variables	7	Clearly define all outcomes, exposures, predictors, potential confounders, and effect modifiers. Give diagnostic criteria, if applicable	5
Data sources/ measurement	8*	For each variable of interest, give sources of data and details of methods of assessment (measurement). Describe comparability of assessment methods if there is more than one group	*6*
Bias	9	Describe any efforts to address potential sources of bias	9
Study size	10	Explain how the study size was arrived at	5
Quantitative variables	11	Explain how quantitative variables were handled in the analyses. If applicable, describe which groupings were chosen and why	NA
Statistical methods	12	(*a*) Describe all statistical methods, including those used to control for confounding	6
		(*b*) Describe any methods used to examine subgroups and interactions	6
		(*c*) Explain how missing data were addressed	Fig 11
		(*d*) If applicable, describe analytical methods taking account of sampling strategy	NA
		(*e*) Describe any sensitivity analyses	NA
**Results**			
Participants	13*	(a) Report numbers of individuals at each stage of study—eg numbers potentially eligible, examined for eligibility, confirmed eligible, included in the study, completing follow-up, and analysed	6
		(b) Give reasons for non-participation at each stage	6
		(c) Consider use of a flow diagram	
Descriptive data	14*	(a) Give characteristics of study participants (eg demographic, clinical, social) and information on exposures and potential confounders	6
		(b) Indicate number of participants with missing data for each variable of interest	6
Outcome data	15*	Report numbers of outcome events or summary measures	6

For peer review only

Main results	16	(*a*) Give unadjusted estimates and, if applicable, confounder-adjusted estimates and their precision (eg, 95% confidence interval). Make clear which confounders were adjusted for and why they were included	7
		(*b*) Report category boundaries when continuous variables were categorized	NA
		(*c*) If relevant, consider translating estimates of relative risk into absolute risk for a meaningful time period	NA
Other analyses	17	Report other analyses done—eg analyses of subgroups and interactions, and sensitivity analyses	7
**Discussion**			
Key results	18	Summarise key results with reference to study objectives	8
Limitations	19	Discuss limitations of the study, taking into account sources of potential bias or imprecision. Discuss both direction and magnitude of any potential bias	9
Interpretation	20	Give a cautious overall interpretation of results considering objectives, limitations, multiplicity of analyses, results from similar studies, and other relevant evidence	9
Generalisability	21	Discuss the generalisability (external validity) of the study results	9
**Other information**			
Funding	22	Give the source of funding and the role of the funders for the present study and, if applicable, for the original study on which the present article is based	10

*Give information separately for exposed and unexposed groups.

Note: An Explanation and Elaboration article discusses each checklist item and gives methodological background and published examples of transparent reporting. The STROBE checklist is best used in conjunction with this article (freely available on the Web sites of PLoS Medicine at http://www.plosmedicine.org/, Annals of Internal Medicine at http://www.annals.org/, and Epidemiology at http://www.epidem.com/). Information on the STROBE Initiative is available at www.strobe-statement.org.

For peer review only
